# Metal ion-responsive nanocarrier derived from phosphonated calix[4]arenes for delivering dauricine specifically to sites of brain injury in a mouse model of intracerebral hemorrhage

**DOI:** 10.1186/s12951-020-00616-3

**Published:** 2020-04-19

**Authors:** Mingxin Li, Guohao Liu, Kaixuan Wang, Lingfeng Wang, Xiang Fu, Lee Yong Lim, Wei Chen, Jingxin Mo

**Affiliations:** 1 Clinical Research Center for Neurological Diseases of Guangxi Province, The Affiliated Hospital of Guilin Medical University, Guilin, 541001 China; 2 School of Pharmacy, Guilin Medical University, Guilin, 541001 China; 3 School of Chemistry, University of New South Wales Sydney, Kensington, NSW 2052 Australia; 4 Division of Pharmacy, School of Allied Health, University of Western Australia, Perth, WA 6009 Australia; 5 Department of Radiology, Affiliated Hospital of Jilin Medical University, Jilin, 132013 China; 6 Department of Pharmacy, The Affiliated Hospital of Guilin Medical University, Guilin, 541001 China

**Keywords:** Intracerebral hemorrhage, Dauricine, Phosphonated calix[4]arene derivative, Targeted delivery, Apoptosis, Ferroptosis

## Abstract

Primary intracerebral hemorrhage (ICH) is a leading cause of long-term disability and death worldwide. Drug delivery vehicles to treat ICH are less than satisfactory because of their short circulation lives, lack of specific targeting to the hemorrhagic site, and poor control of drug release. To exploit the fact that metal ions such as Fe^2+^ are more abundant in peri-hematomal tissue than in healthy tissue because of red blood cell lysis, we developed a metal ion-responsive nanocarrier based on a phosphonated calix[4]arene derivative in order to deliver the neuroprotective agent dauricine (DRC) specifically to sites of primary and secondary brain injury. The potential of the dauricine-loaded nanocarriers for ICH therapy was systematically evaluated *in vitro* and in mouse models of autologous whole blood double infusion. The nanocarriers significantly reduced brain water content, restored blood-brain barrier integrity and attenuated neurological deficits by inhibiting the activation of glial cells, infiltration by neutrophils as well as production of pro-inflammatory factors (IL-1β, IL-6, TNF-α) and matrix-metalloprotease-9. These results suggest that our dauricine-loaded nanocarriers can improve neurological outcomes in an animal model of ICH by reducing inflammatory injury and inhibiting apoptosis and ferroptosis.

## Introduction

Primary intracerebral hemorrhage (ICH) is the most devastating type of stroke [1]. It affects 2 million people worldwide each year and is associated with high disability and mortality rates, which have not changed substantially for decades. Individuals who suffer ICH have poor prognosis mainly because of secondary brain injuries after the stroke, including hematoma toxicity, oxidative stress and inflammatory injury [2]. Much secondary injury arises from neuroinflammation: hematoma activates glial cells, which disrupt the blood-brain barrier, allowing infiltration by peripheral inflammatory cells that produce abundant cytokines, which in turn stimulate neuronal apoptosis and impair neurological function [3, 4]. Another major cause of secondary injury is ferroptosis due to the abundant free iron released from lysed erythrocytes in stroke-injured brain tissue [5, 6]. The complexity of ICH helps explain why no specific treatment exists, despite the burden it places on health systems worldwide.

**Scheme 1:**
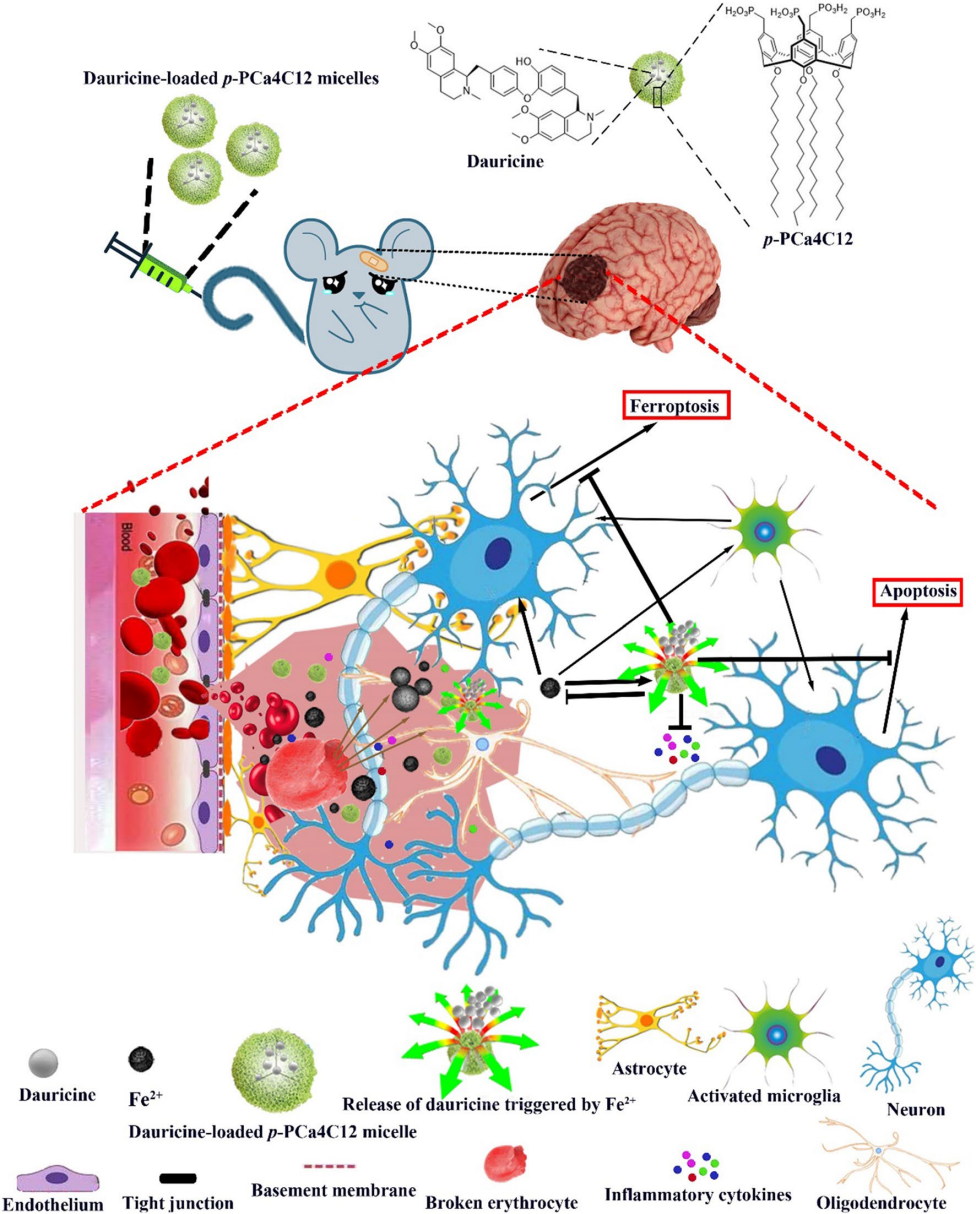
Schematic of the composition and activating environment of metal ion-responsive nanocarriers based on a phosphonated calix[4]arene derivative loaded with the neuroprotective agent dauricine. The micelles release dauricine in response to abundant Fe^2+^ at sites of primary and secondary brain injury. The present study was carried out in a mouse model of ICH

A neuroprotective agent that may help mitigate secondary stroke injury is the isoquinoline alkaloid dauricine (DRC), isolated from the Chinese herbal medicine Rhizoma Menispermi (Scheme 1) [7]. DRC can protect the brain from ischemic damage by up-regulating Bcl-2 and down-regulating Bax expression, thereby inhibiting neuronal apoptosis, as well as by stimulating IRE-1/XBP-1 signaling and down-regulating caspase-3, which relieves endoplasmic reticulum stress [8]. However, DRC on its own shows poor oral absorption, high rate of metabolism and rapid systemic elimination, which means its actual concentration at hemorrhagic sites is very low [9].

We hypothesized that we could couple DRC with an appropriate carrier that would be responsive to metal ions such as Fe^2+^ that are more abundant at hematoma sites than in normal tissue. As a drug delivery system, we focused on the calixarene macrocyle and its derivatives, which have antiviral, antibacterial, antifungal, antitubercular, anticancer, and anti-oxidant activities [10–13]. We have shown that a *p*-methylenebisphosphonic calix[4]arene derivative can assemble into micelles to form host-guest complexes with small molecules in the cavity, such as the drug carboplatin [14, 15]. Furthermore, calixarene derivatives can form host–guest complexes with free metal ions such as Cu^2+^ and Fe^2+^, in some cases with metal selectivity [16–19]. We envisioned that using calixarene derivatives loaded with DRC could synergistically treat ICH by binding selectively to metal ions and by releasing DRC selectively at hemorrhagic sites. In the present study, we designed *O*-dodecyl *p*-methylenebisphosphonic calix[4]arene micelles containing DRC (hereafter referred to as DPM) and explored their anti-inflammatory and neuroprotective effects in a mouse model of ICH (Scheme 1).

## Materials and methods

### Reagents

All reagents, solvents, chemicals and plastic cell culture supplies were obtained from Sigma-Aldrich (St. Louis, MO, USA) or Fisher (Pittsburgh, PA, USA) unless otherwise mentioned. Annexin V-FITC/PI Apoptosis Kits were purchased from Lianke Technology (Hangzhou, China). Matrigel^®^ Basement Membrane Matrix was obtained from Corning (NY, USA). Ammonium ferrous sulfate was ordered from Macklin (Shanghai, China). ROS Assay Kit was obtained from Beyotime (Shanghai, China). DRC was obtained from Aladdin Chemical Reagent Co., Ltd (Shanghai, China). Paraformaldehyde (4%) was purchased from Guangzhou Ruishu Biotechnology Co., Ltd (Guangzhou, China). Ultrapure deionized water was obtained from a Millipore system (resistivity, 18.2 MΩ cm). Phosphonato calixarene (*p*-PCa4C12, purity > 95%) was synthesized in our laboratory according to published methods with some minor modifications. Its chemical structure was confirmed by ^1^H-NMR (Varian Mercury 400, USA; Additional file 1: Figure S1).

### Cells

Human SH-SY5Y neuroblastoma cell lines (Cell Resource Centre, Guilin Medical University, China) were cultured in complete medium (Dulbecco’s modified Eagle’s medium, DMEM) supplemented with 10% fetal bovine serum (FBS; Gibco, Invitrogen, Shanghai, China) Cultures were maintained in a humidified 5% CO_2_ atmosphere.

### Animals and groups

**Fig. 1:**
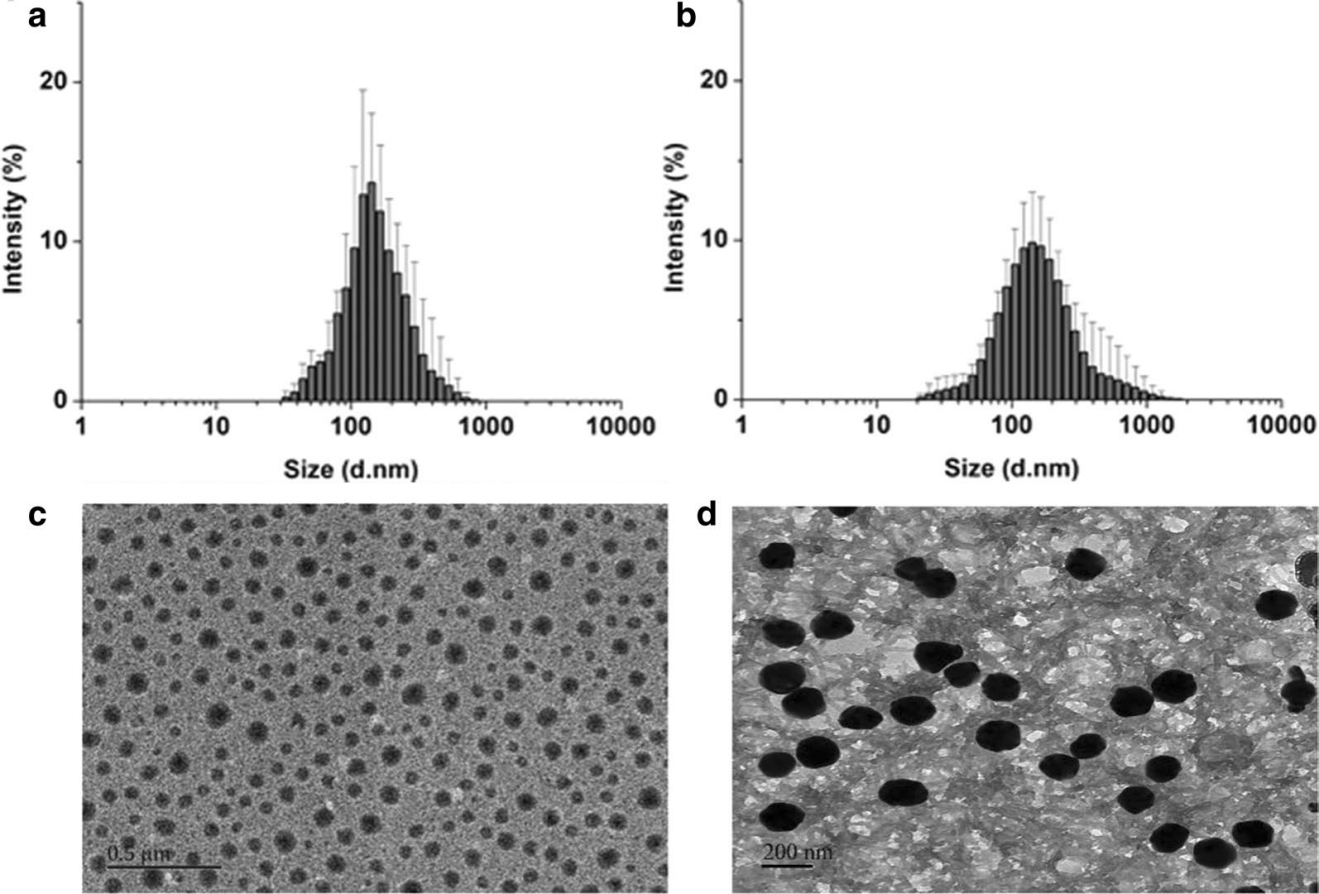
Characterization of size and morphology of blank *p*-PCa4C12 micelles (PM) and dauricine-loaded *p*-PCa4C12 micelles (DPM). Dynamic light scattering was used to analyze the size distribution of **a** PM and **b** DPM, while transmission electron microscopy was used to examine morphology of **c** PM, Scale bar, 0.5 μm and **d** DPM, Scale bar, 200 nm. n = 5

Animal protocols were approved by the Department of Laboratory Animal Research at Guilin Medical University (License No. YXLL-2017-167) in compliance with the Principles of Laboratory Animal Care (People’s Republic of China). Adult male C57BL/6 mice weighing 20 to 28 g were used in this study. Mice were maintained at a constant ambient temperature (22 ± 1 °C) on a 12-h light/dark cycle. Mice were randomly divided into the following five groups based on random numbers generated using SPSS (IBM, Chicago, IL, USA). The sham group (n = 24, of which 24 survived) was subjected to mock surgery (craniotomy without blood infusion) and treated with 0.1 mL 0.9% saline. The vehicle group (n = 26, of which 21 survived) was subjected to ICH surgery, then treated with 0.9% saline. The DRC group (n = 24, of which 21 survived) was subjected to ICH surgery, then immediately treated with 10 mg/kg DRC (Sigma, purity ≥ 95%, Fig. 1) via tail vain injection. The drug was dissolved in 0.05 mL 20.0% (v/v) HCl, then neutralized with NaOH (Sigma, USA, purity ≥ 99.0%). The PM group (n = 24, of which 20 survived) was subjected to ICH surgery, then treated with 90 mg/kg empty *p*-PCa4C12 micelles (PM). The DPM group (n = 24, of which 22 survived) was subjected to ICH surgery, then treated with 100 mg/kg dauricine-loaded *p*-PCa4C12 micelles (DPM, equivalent to 10.1 mg/kg DRC).

### Fabrication of PM and DPM

DPM were fabricated by a thin-film hydration method. In brief, 100 mg of *p*-PCa4C12 and 30 mg of DRC were dissolved in 50 mL of chloroform in a 150-mL round-bottom flask. The flask was attached to a rotary evaporator (BUCHI, Switzerland) to get rid of chloroform overnight in a 37 °C water bath. The resultant thin film was rehydrated at 37 °C for half an hour in 20 mL of deionized water and then sonicated (probe sonicator, 50% strength; Q700 of QSonica^®^, Newtown, CT, USA) for 5 min to form DPM. Insoluble material was eliminated using a 0.22-μm filter (Millipore filter), and the filtered DPM were freeze-dried and stored at − 20 °C for further experiments. The same procedure but without DRC was used to prepare PM.

### Physical characterization of micelle formulations

Particle size, polydispersity index (PDI), and zeta potential of DPM and PM were investigated using dynamic light scattering (Malvern Nano ZS90 laser particle size analyzer, Malvern, UK). Morphology of PM and DPM was analyzed using transmission electron microscopy (JEOL, Japan).

### In vitro release profiles and stability of DPM in different Fe^2+^ concentrations

Lyophilized DPM was precisely weighed and dispersed in release medium [phosphate-buffered saline (PBS, pH 7.4) with or without 5 mM ammonium ferrous sulfate] to obtain a final DRC concentration of 2 μM. DPM solution (5 mL) was placed into a sealed dialysis bag (Spectrum Laboratories, Rancho Dominguez, CA, USA) with a molecular weight cut-off of 1000 Da, then transferred to 95 mL of phosphate-buffered saline (PBS, pH 7.4) containing 0.5% Tween 80 with or without 5 mM Fe^2+^ (ammonium ferrous sulfate). The system was incubated at 37 ± 0.5 °C with stirring at 100 rpm. At specific intervals, the medium containing the dialysis bag was sampled (0.5 mL), then fresh 0.5 mL release medium was replaced, and DRC was assayed using HPLC (Shimadzu LC-20A) as described in *Supplementary Information*. All procedures, including incubation and HPLC detection, were carried out under minimal light conditions.

### Neuroprotective effects of DPM

SH-SY5Y cells were seeded in 96-well plates in 100 μL of DMEM medium (1 × 10^4^ cells/well) and incubated for 24 h. Free DRC, PM or DPM was added to wells at a series of concentrations (0.01 μM–1 μM), with all wells except for negative control group also receiving ammonium ferrous sulfate to a final concentration of 5 mM. Cells were incubated another 24 h at 37 °C and cell viability was tested using the CCK-8 assay.

### Assay of intracellular reactive oxygen species (ROS)

Intracellular ROS generation in SH-SY5Y cells was detected using the ROS Assay Kit. Typically, SH-SY5Y cells were seeded into a 35-mm glass-bottom culture dish (NEST, China) at a density of 1 × 10^6^ cells/well and cultured overnight in 2 mL DMEM medium with 10% FBS at 37 °C. The medium was replaced with fresh DMEM and 10% FBS with or without Fe^2+^ at a final concentration of 5 mM, followed by addition of 1 µM DRC, 5 µM PM or DPM to a final DRC concentration of 1 µM. Negative control cultures were not exposed to Fe^2+^, free DRC or micelles. The vehicle group was treated with Fe^2+^ and then 0.1 mL DMSO. After 12-h incubation, the medium was replaced with fresh DMEM (2 mL) containing ROS reagent (10 μM), then cells were incubated another 20 min, washed three times with ice-cold PBS and imaged and semi-quantified with fluorescent confocal microscopy (Leica TCS SP5, Germany). The extent of ROS-mediated decomposition of 2’,7’-Dichlorodihydrofluorescein diacetate (DCFH-DA) into dichlorofluorescein (DCF) was measured at an excitation wavelength of 488 nm and emission wavelengths of 500 to 540 nm. Three fields of view were randomly chosen for each group before Image-Pro Plus 5.1 software (Media Cybernetics Inc., Rockville, MD, USA) was used for quantitative analysis of ROS (area × intensity).

### Apoptosis detection based on Annexin V / propidium iodide staining

SH-SY5Y cells were cultured for 12 h in the presence of 5 mM Fe^2+^ (to induce apoptosis) as well as 1 µM DRC, 5 µM PM or DPM (final DRC concentration, 1 µM). Then the medium was replaced with fresh DMEM and the cells were incubated another 24 h. Cells were trypsinized, collected by centrifugation at 300*g* for 5 min, rinsed twice with PBS and resuspended in 500 μL of binding buffer. The cells were mixed with 5 μL of Annexin V-FITC and propidium iodide (PI), then the suspension was mixed and incubated in the dark for 15 min. Cell apoptosis was analyzed by flow cytometry (Becton Dickinson, Franklin Lake, NJ, USA).

### Mouse model of autologous blood ICH

ICH was induced using the autologous whole blood double infusion model (30 μL total infusion). Mice (5-week-old male; Orient Bio, Hunan, China) were anesthetized with continuous isoflurane (4% induction, 1.5–2% maintenance) and immobilized on a stereotactic frame (Stoelting, Wood Dale, IL, USA). After making a small midline sagittal incision in the skin overlying the skull, a craniotomy was performed 0.5 mm anterior and 2.4 mm right relative to the bregma [20, 21] (Additional file 1: Figure S2). Autologous blood was collected onto a sterile surface by needle prick of the tail artery after first cleaning the area with 70% ethanol and gently warming the tail for 2 min with a heat lamp. Blood was immediately drawn into PE-20 tubing (Instech, Plymouth Meeting, PA) connected on one side to a 50 mL syringe with a 26-gauge luer tip needle (Hamilton Company, Reno, NV, USA) located within an automated injector, and the other side to a 26-gauge needle with the beveled end inserted into the tubing [21]. The blunt end of this needle was inserted 3.9 mm ventral from the skull surface, relocated to 3.6 mm, and left in place for 7 min. After the waiting period, 10 μL of blood was infused, followed by an additional 5 min waiting period prior to a second infusion of 20 μL [22]. All injections were performed at 1.0 μL/min using an automated injector (Stoelting, Wood Dale, IL, USA). The needle was left in place for 10 min after the second infusion, then slowly removed over a 25-min period. The sham model was set up by performing craniotomy without blood infusion.

### Behavior testing

Two trained investigators (Lingfeng Wang and Kaixuan Wang) who were blinded to group allocations determined the modified neurological severity score (mNSS) at 24 h after ICH. This assessment of neurological deficit evaluates abnormal movements as well as motor, sensory, and reflex deficits, which are scored on an 18-point neurological deficit scale. Maximal deficit score was 18, with higher scores indicating worse deficit. Eight animals were randomly chosen from each group for evaluation.

### Brain water content measurement

Brain water content was measured after ICH as previously described [23]. Brains (n = 8) were harvested 24 h after ICH, and 4-mm-thick coronal brain samples were collected from 2 mm anterior and 2 mm posterior to the injection site. The brains were divided into 2 parts: ipsilateral and contralateral hemisphere. Brain samples were immediately weighed to obtain the wet weight and then dried at 70 °C for 48 h to obtain the dry weight. Brain water content (%) was calculated as (wet weight − dry weight)/wet weight × 100%.

### Assessment of blood–brain barrier permeability

The Evans blue extravasation test was performed to examine the permeability of the blood-brain barrier as previously described [24]. Evans blue (2%, 4 mL/kg, Sigma, USA) was injected into the tail vein of the mice at 24 h after ICH. After 2 h, mice (n = 5–6) were euthanized and perfused with 250 mL saline. Brain hemispheres were quickly removed and weighed. Each sample was incubated in trichloroacetic acid, homogenized and centrifuged. The supernatant was diluted with ethanol. The absorbance of the supernatant solution at 620 nm was measured using a spectrophotometer (Thermo Fisher Scientific, USA) and quantified based on a standard curve.

### Ex vivo imaging of micelle accumulation in the brain

For *ex vivo* imaging studies, after the ICH mouse model was set up, groups of C57BL/6 mice were injected with 1 µg/g of the near-infrared dye DiR (1,1′-Dioctadecyl-3,3,3′,3′-Tetramethylindotricarbocyanine Iodide) on its own or loaded into PM (DiPM). Stock solutions were prepared as 1 mg DiR in 100 mL 0.9% saline or an equivalent amount of DiPM in 100 mL 0.9% saline. At 12 and 24 h of injection, organs were then excised from mice and imaged with an IVIS Spectrum imaging system (Caliper, USA) with excitation at 748 nm and absorption at 780 nm in order to capture the fluorescence emitted by DiR.

### MRI image acquisition

Data were obtained on a SIEMENS Verio 3.0 Tesla MRI scanner (Siemens Healthineers, Erlangen, Germany) using a mouse magnetic resonance coil (WanKang, Wuxi, China). All images were analyzed by experienced radiologists who were blinded to the animal treatment. The imaging protocol for all mice included a T2 fast spin-echo. The field of view was 20 × 20 mm, and the matrix was 256 × 256 mm. Seven coronal slices (1 mm thick) were acquired from the frontal pole to the brain stem, and the images were saved as images of 256 × 256 pixels.

### Histology and quantification

Histology and quantification were conducted as previously described [25] with minor modifications. Briefly, 10 sets of 16 sections equally distributed throughout the hematoma and anteroposterior brain regions were processed. The tissue blocks were cut into 4-μm sections and stained with hematoxylin and eosin to assess pathological changes in the brain. Cresyl violet staining was used to assess lesion and hematoma volume, tissue injury, percent ipsilateral hemispheric enlargement, and ventricular volume. Perls’ iron staining was used to evaluate iron deposition. Photomicrographs were acquired using a light microscope (Nikon ECLIPSE 80i, Nikon, Tokyo, Japan).

### Western blot analysis

Brain samples were obtained at 24 h after ICH. Protein from perihematomal tissue (n = 4–6) was extracted and analyzed according to our previous study [26]. In brief, the brain sample was homogenized and centrifuged. An equal amount of protein from each sample was mixed with loading buffer, denatured, separated on an 8–12% (v/v) SDS gel, and transferred to nitrocellulose membranes. Membranes were blocked with 5% nonfat dry milk and incubated with primary antibody against the following proteins: GPX4 (ab41787, Abcam), caspase-3 (ab90437), MMP9 (sc-6841, Santa Cruz Biotechnology), ZO-1 (sc-8147), β-actin (sc-47778), Bcl-2 (sc-783), and Bax (sc-6236). Then membranes were treated with horseradish peroxidase-conjugated anti-rabbit/mouse IgG secondary antibody, detected with a chemiluminescence substrate (Thermo Scientific) and visualized using the ChemiDoc™ MP Imaging System (Bio-Rad). The density of blot bands was quantified using ImageJ software (NIH, USA).

### Elisa

Perihematomal brain tissue (n = 5–6) was collected at 24 h after ICH, homogenized and centrifuged. The supernatant was assayed for IL-1β, IL-6 and TNF-α using enzyme-linked immunosorbent assays (ELISAs) according to the manufacturer’s instructions (catalog nos. H002, H007, H052, Jiancheng Bioengineering Institute, Nanjing, China). Concentrations were represented as pg/mL.

### Immunofluorescence

Brain cryosections (10 μm) from 4–5 animals in each group were blocked with 3% bovine serum albumin and then incubated with 1:100 dilutions of primary antibodies against the following proteins: GFAP (16825-1-AP, Proteintech, USA), Iba-1 (ab5076, Abcam), MPO (ab9535) and ZO-1 (21773-1-AP, Proteintech). Nuclei were stained with DAPI. Immunofluorescence was observed using a BX51 fluorescence microscope (Olympus, Tokyo, Japan).

### Statistical analysis

Data were expressed as mean ± standard deviation (SD). Statistical differences were evaluated using ANOVA with post hoc Bonferroni test for groups comparison. The researchers performing statistical analysis were blinded to the treatment conditions for each dataset. All measurements were made in triplicate unless otherwise mentioned, and all experiments were performed twice. *P < 0.05, **P < 0.01 and ***P < 0.001.

## Results

### Particle size, zeta potential, morphology, and encapsulation efficiency of PM

The particle sizes of the PM and DPM were determined via DLS. The average diameter of PM was 123.3±18.0 nm at room temperature (Fig. 1a), which increased to 186.6 ± 16.5 nm after encapsulation of DRC (Fig. 1b). DRC loading also changed the zeta potential of PM from − 19.33 ± 1.59 to − 15.21 ± 3.68 mV (Table 1). The polydispersity index (PDI) was 0.193 ± 0.028 for PM and 0.232 ± 0.086 for DPM, both of which are below 0.3 and therefore indicate narrow particle size distribution.

**Table 1:** Characterizations of blank micelles (PM) and dauricine-loaded micelles (DPM)

Sample	Diameter (nm)^a^	Polydispersity index^a^	Zeta potential (mV)^a^	Encapsulation efficiency (%)^b^	Drug loading (%)^b^
PM	123.3 ± 18.0	0.193 ± 0.028	− 19.33 ± 1.59	NA	NA
DPM	186.6 ± 16.5	0.202 ± 0.086	− 15.21 ± 3.68	97. 10 ± 5. 21	10.10 ± 1.31

*NA* not applicable

n = 3, mean ± SD

^a^Based on dynamic light scattering

^b^Based on HPLC

Transmission electron microscopy images showed that PM were uniformly spherical in shape and homogeneous in size (Fig. 1c), while size distribution of DPM micelles became larger after loaded with DRC (Fig. 1d), in line with the DLS data. Encapsulation efficiency (EE) and drug loading (DL) were calculated, respectively, as 97.10 ± 5.21% and 10.10 ± 1.31% (Additional file 1: Figure S3).

### In vitro release of DRC from DPM

**Fig. 2:**
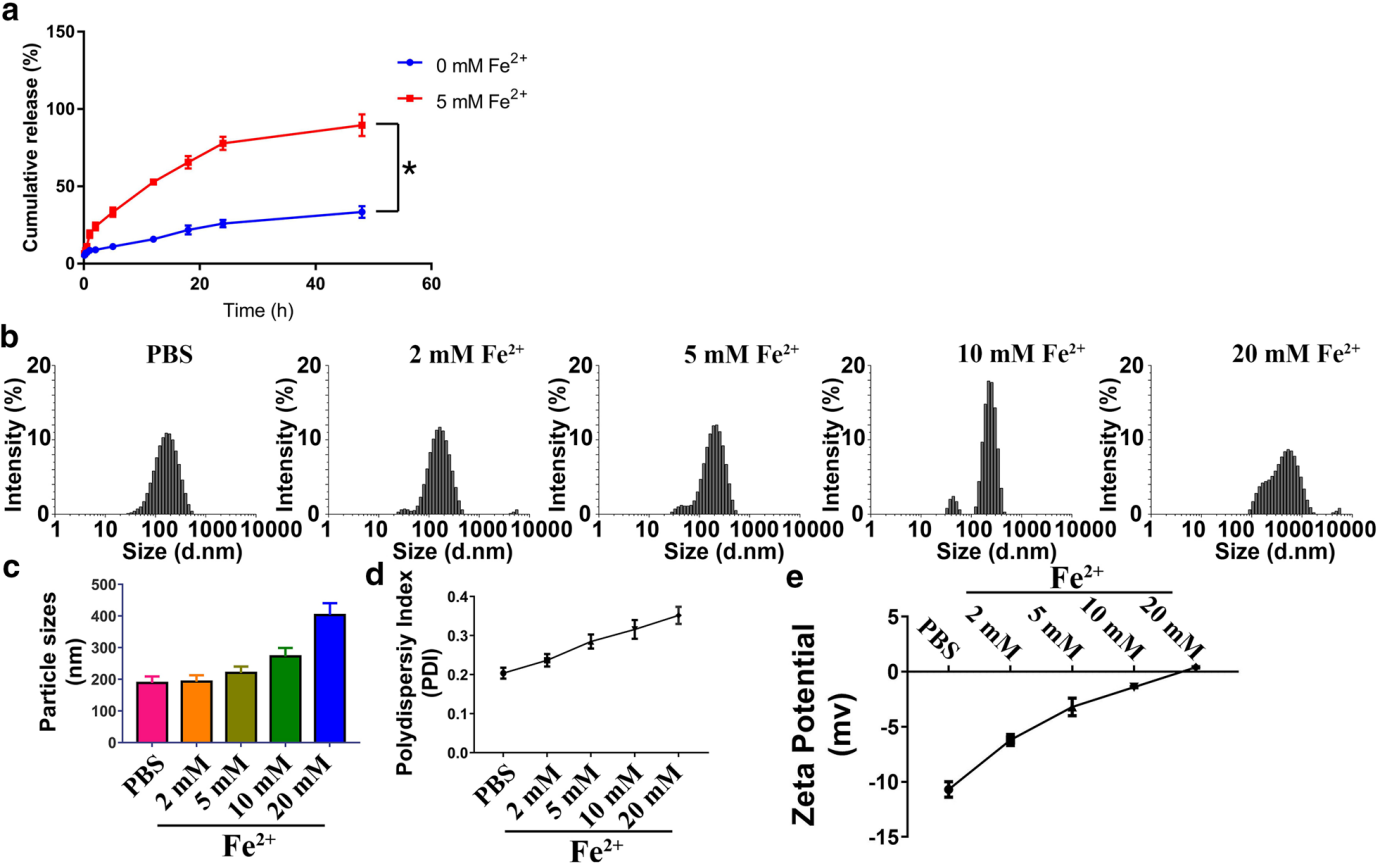
Release profile of dauricine (DRC) from DRC-loaded micelles (DPM) in PBS buffer **a** with or without 5 mM Fe^2+^ over 48 h. *P < 0.05. Changes in the following DPM parameters were measured after 24 h incubation in PBS, and in PBS with increasing concentrations of Fe^2+^: **b** size distribution, **c** mean diameter, **d** polydispersity index (PDI) and **e** zeta potential. Results are mean ± SD (n = 3)

The DRC release profiles from DPM in PBS (pH 7.4) with or without 5 mM Fe^2+^ are shown in Fig. 2a. DPM was stable in PBS without Fe^2+^, showing only about 25% release of DRC after 24 h at 37 °C. In the presence of 5 mM Fe^2+^, in contrast, more than 24% of the DRC load was released from DPM within 2 h and about 80% by 24 h. DPM maintained a stable mean diameter of 189.8 ± 20.5 nm and narrow size distribution during 24-h incubation in PBS (Fig. 2b), which did not change substantially for at least 96 h (Additional file 1: Figure S4). These results indicate that DPM can remain intact for long periods in the absence of Fe^2+^, and then release DRC selectively in response to high Fe^2+^ concentrations. It has been proposed that Fe^2+^ can neutralize the anionic head groups of phosphonates in PM, destabilizing the micelles and accelerating DRC release. Consistent with this idea, we found that adding Fe^2+^ to PM or DPM increased mean micelle size, polydispersity index and zeta potential (Fig. 2c–e).

### Neuroprotective effects of DPM

**Fig. 3:**
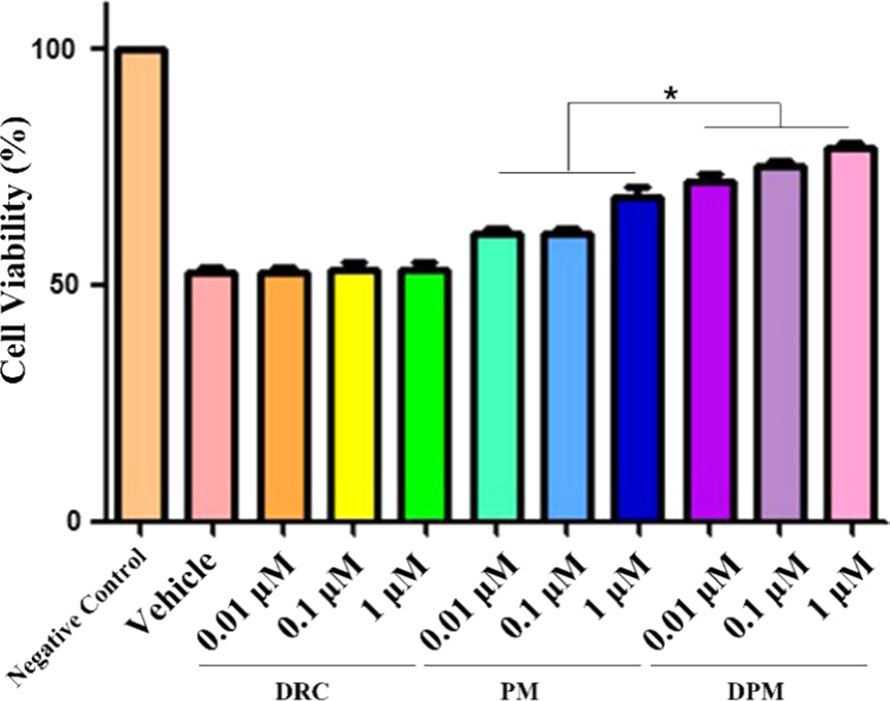
Neuroprotective effects of DPM against toxicity of 5 mM Fe^2+^ in SH-SY5Y cells. Cells were treated with free DRC, blank micelles (PM) or dauricine-loaded micelles (DPM). *P < 0.05 (n = 5)

The therapeutic potential of DPM was analyzed *in vitro*, based on studies implicating massive release of metal ions such as Fe^2+^ and high levels of neurotoxic ROS in the neuronal cell injury and apoptosis of ICH [27]. Figure 3 and Additional file 1: Figure S5 show the protective potential of different micelle formulations against Fe^2+^- and Cu^2+^-mediated effects on SH-SY5Y cells. Addition of 5 mM Fe^2+^ reduced SH-SY5Y cell viability by about 50% (Additional file 1: Figure S6), while free DRC could only slightly reverse this, DPM significantly increased the cell viability (Fig. 3), with assistance of good biocompatible PM (Additional file 1: Figure S7). Similar results were observed with the addition of Cu^2+^ (Additional file 1: Figure S5).

### Ability of DPM to inhibit ROS production

**Fig. 4:**
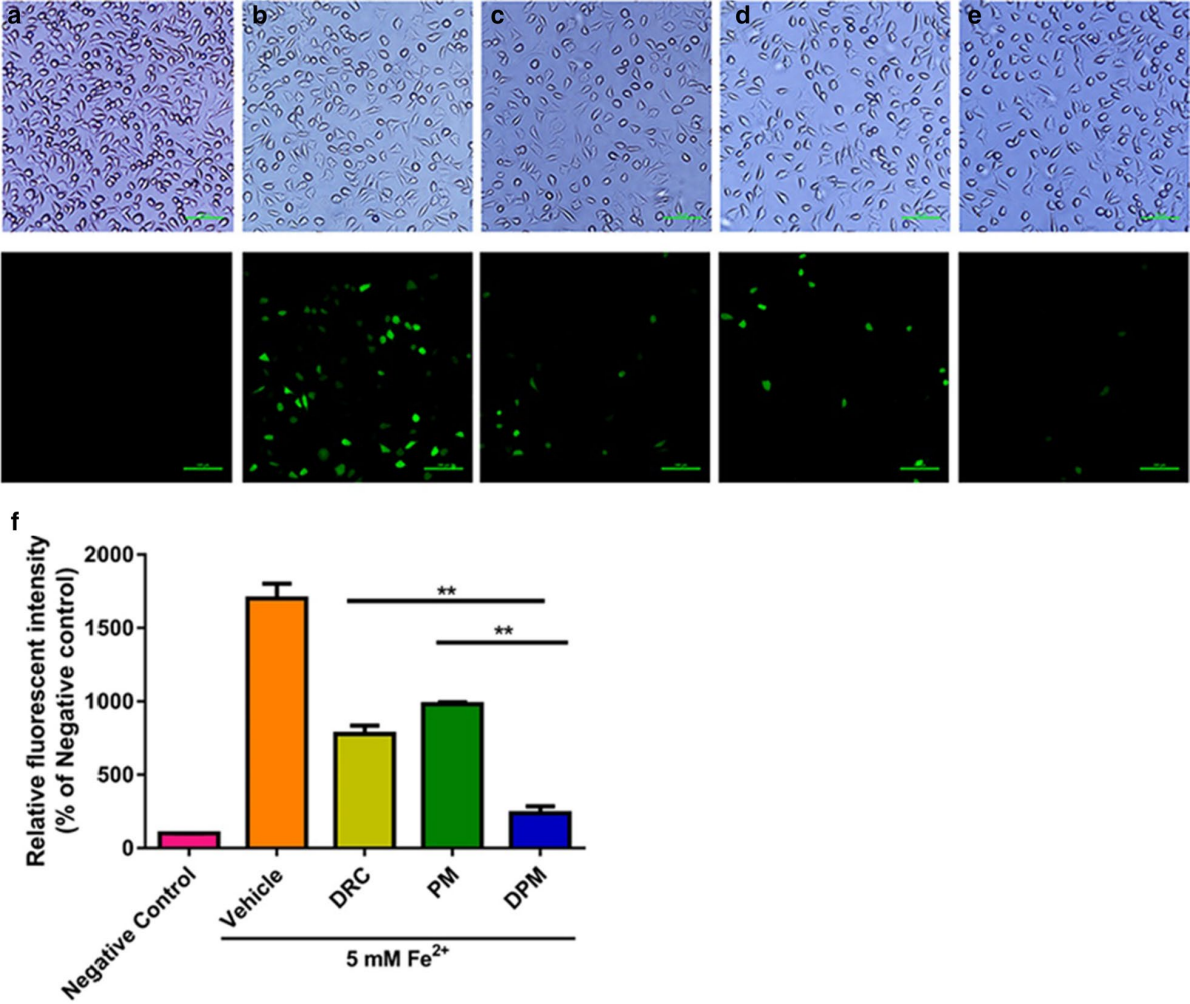
Representative micrographs of SH-SY5Y cultures treated with **a** nothing (cell culture medium only), **b** vehicle (DMSO), **c** DRC (1 μM), **d** PM (5 μM), or **e** DPM (1 μM). **f** Histogram of intracellular ROS levels after exposure to the various formulations. Scale bar 100 µm **P < 0.01 (n = 3)

We compared the ability of free DRC, PM and DPM to reduce Fe^2+^-induced intracellular ROS generation, measured as DCF fluorescence (Fig. 4). ROS production was lower with any of the three treatments than with vehicle, and it was lowest with DPM (7.2-fold lower than vehicle). This large inhibition of ROS production may reflect the ability of DPM not only to protect neurons but also to chelate the toxic Fe^2+^.

### Ability of DPM to inhibit apoptosis

**Fig. 5:**
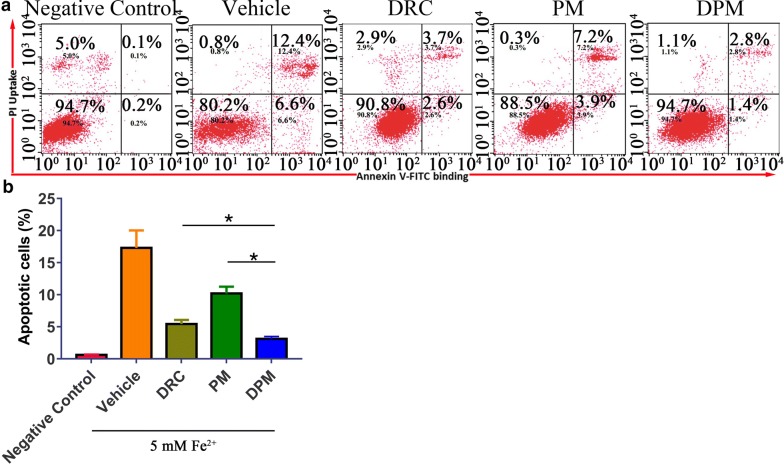
**a** Flow cytometry of SH-SY5Y cells treated first with Fe^2+^ to induce apoptosis, and then with vehicle (DMSO), free dauricine (DRC, 1 μM), blank micelles (PM, 5 μM) or dauricine-loaded micelles (DPM, 1 μM). Negative control group was treated with cell culture medium only without Fe^2+^, Cells were stained with annexin V-FITC and propidium iodide, then sorted by flow cytometry. **b** Percentage of apoptotic cells in different treatment groups. *P<0.05 (n=5)

Apoptosis was induced in SH-SY5Y cells by 5 mM Fe^2+^, which led to 17.37 ± 2.73% of apoptotic cells. Treatment with 1 μM DPM reduced this fraction to 3.11 ± 0.37%, lower than the fraction of 5.43 ± 0.64% with free DRC and much lower than the fraction of 10.19 ± 1.06% with PM (Fig. 5).

### Ability of DPM to improve ICH-induced neurological deficits

**Fig. 6:**
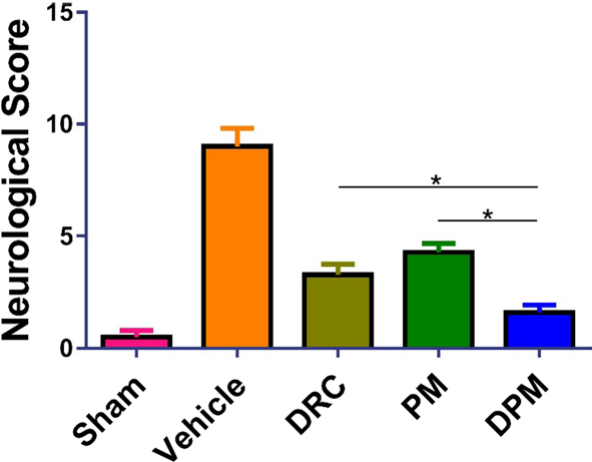
Assessment of neurological defects in mice at 24 h after ICH and treatment with vehicle (0.9% saline), free dauricine (DRC), blank micelles (PM) or dauricine-loaded micelles (DPM). The sham group was set up by performing craniotomy without blood infusion. Animals were scored using the modified neurological severity score. Results are mean ± SD (n=10 per group). *P<0.05

Before ICH, mNSS was 0 in all animals, and the inducement of ICH significantly increased (worsened) this score. Treatment for 24 h with DPM led to a significantly lower score than treatment with DRC (7.63 ± 1.30 vs 9.50 ± 1.41, p < 0.05; Fig. 6). In addition, DPM animals scored better than any other group of ICH animals in paw placement test and corner turn test (Additional file 1: Figure S8).

### Ability of DPM to alleviate brain edema and loss of blood–brain barrier permeability after ICH

**Fig. 7:**
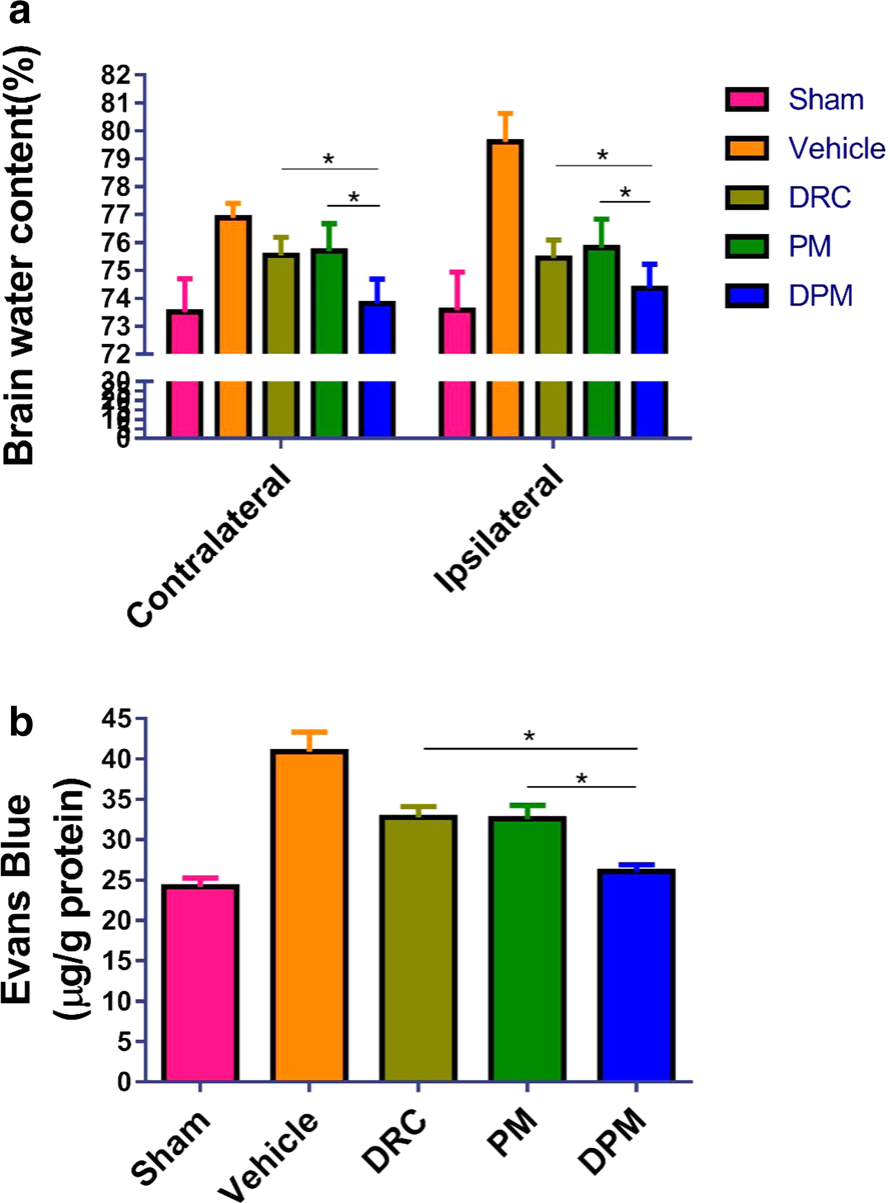
Assessment of the ability of DPM to reduce brain water content and reverse Evans blue leakage in ipsilateral and contralateral brain hemispheres at 24 h after ICH. **a** Brain water content (n=5). **b** Evans blue extravasation assay. Sham group, n=6; vehicle group, n = 5; DRC group, n = 6; PM group, n = 5; DPM group, n = 6. Values are mean±SD. * P < 0.05

To evaluate effects of DPM on brain edema after ICH, brain water content was examined in contralateral and ipsilateral cortices following treatment at 24 h after ICH (Fig. 7a). DPM was associated with significantly lower brain water content than free DRC in the contralateral cortex (73.80 ± 1.89% vs 75.55 ± 1.59%, P < 0.05) and ipsilateral cortex (74.36 ± 1.95% vs 75.45 ± 1.61%, P < 0.05).

To investigate the effects of DPM on ICH-induced permeabilization of the blood-brain barrier, an Evans blue extravasation assay was performed at 24 h after ICH. DPM was associated with significantly less Evans blue leakage than free DRC (26.05 ± 1.93 vs 32.77 ± 2.96 µg/g, P < 0.05, Fig. 7b).

Taken together, these results suggest that DPM mitigates ICH-induced neurological deficits, brain edema, and blood-brain barrier permeabilization.

### Accumulation of micelles at hematoma sites

**Fig. 8:**
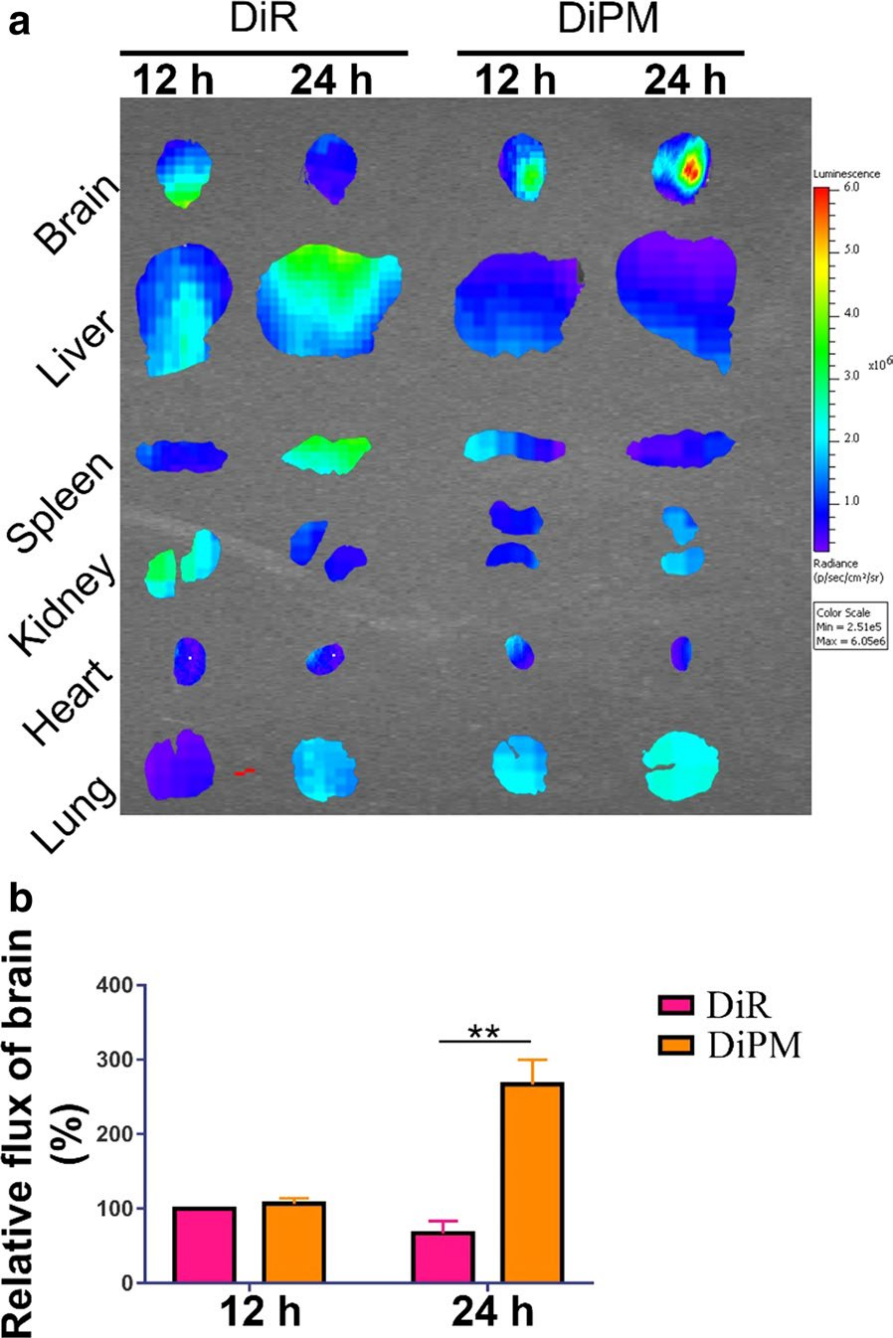
Distribution of DiR-labeled empty micelles (DiPM) in ICH mice, assessed at 12 and 24 h after tail vein injection into mice with ICH. Key organs were removed and examined using an IVIS system. **a** DiPM accumulated at the hemorrhagic site at much higher level than free DiR. Images show the fluorescence intensity as an overlay in accordance with the scale. **b** Quantitation of DiR and DiPM in brain at 12 and 24 h after injection. **P < 0.01 (n=3)

**Fig. 9:**
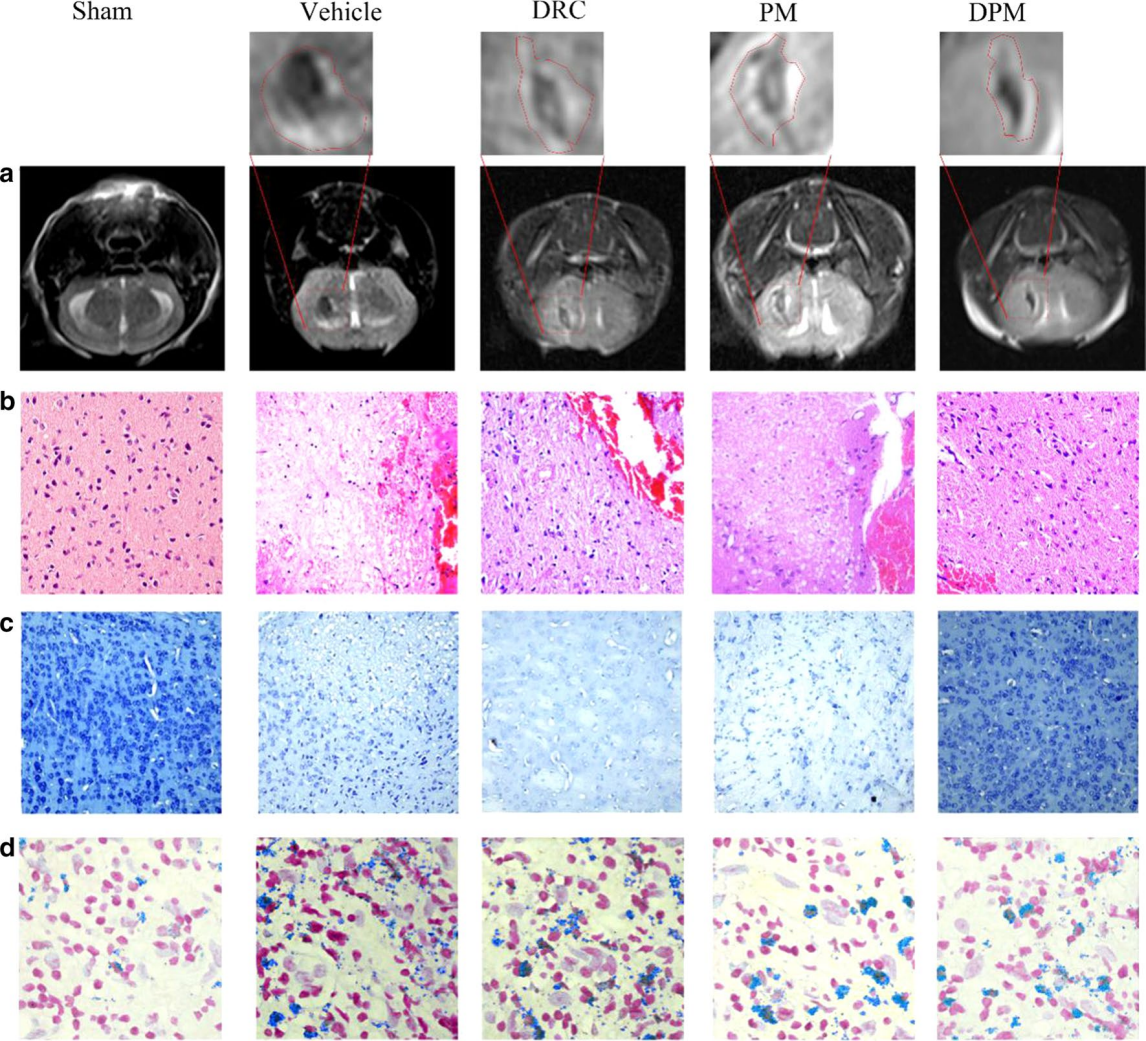
Effect of DPM on ICH-induced brain damage in a mouse model of autologous blood ICH. **a** T2-weighted turbo spin echo magnetic resonance images after 24 h of false ICH operation (sham), 0.9% saline (vehicle), free dauricine (DRC), empty micelles (PM) or dauricine-loaded micelles (DPM). The areas demarcated with dash lines contain a bright rim surrounding the hematoma due to edema formation and/or extruded serum from the injected blood. **b** Representative sections of brain tissue stained with hematoxylin-eosin. Magnification, ×100. **c** Representative sections of brain tissue stained with cresyl violet. Magnification, ×20. **d** Representative sections of brain tissue stained with Perl’s stain to detect iron. Magnification, ×200

We hypothesized that the micelles would preferentially release their cargo at sites of hematoma injury because the negative charges on the micelle surface should be neutralized by abundant metal ions in the surroundings, as confirmed in vitro (Fig. 9d). To test this, we examined the distribution of micelles in ex vivo organs from treated animals at 12 and 24 h post-injection. To enable micelle tracking, we loaded PM with the near-infrared fluorescent dye DiR and injected the micelles into mice via the tail vein immediately after ICH. DiR signals in organs of both cases at different time points were shown in Fig. 8a. The proportion of DiR signal in the brain was much higher in DPM-treated mice than in animals treated with free dye after 24 h (Fig. 8b). These results suggest that, as desired, DPM tends to accumulate, and release its DRC cargo, at sites of ICH injury in the brain.

### Ability of DPM to alleviate ICH-induced brain damage in a mouse model of autologous blood ICH

Mice were subjected to MRI examination following treatment at 24 h after ICH induction. T2-weighted MRI showed obvious brain hematoma, edema and injury in all mice. The MRI signal was mixed and included hypointense signal, suggestive of hematoma tissue, as well as a hyperintense signal surrounded by dark rims, suggestive of perihematoma edema. DPM mitigated these effects of ICH to a greater extent than free DRC (Fig. 9a). These effects of ICH and the neuroprotective effects of DPM were confirmed by staining brain tissues with hematoxylin–eosin and cresyl violet (Fig. 9b, c).

The effects of DPM were associated with a reduction in ferric iron deposition (as blue color indicating), based on Perl’s staining of brain sections (Fig. 9d). Iron deposition is triggered by ICH and induces production of ROS and lipid hydroperoxides to lethal levels, known as ferroptosis [28].

### Ability of DPM to reverse ZO-1 down-regulation

**Fig. 10:**
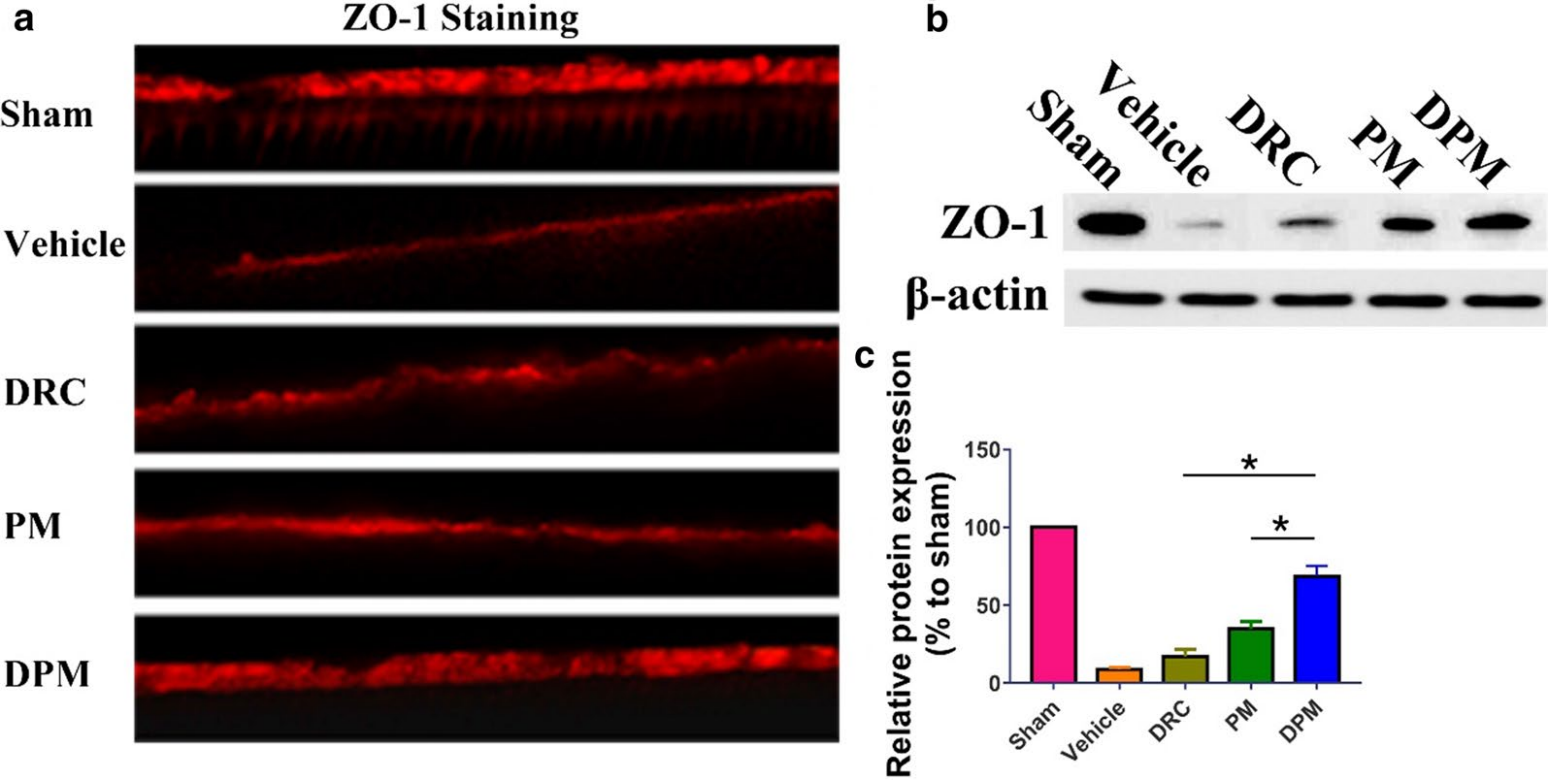
DPM significantly preserved zonula occludens-1 (ZO-1) expression after ICH. **a** Representative photographs of immunostaining (red) for ZO-1 in the perihematomal area at 24 h after ICH. Magnification, ×400. **b** Western blotting of perihematomal tissue. **c** Quantitation of ZO-1. Values are mean ± SD, n = 3. *P < 0.05

We used immunofluorescence staining and western blotting to detect the expression of ZO-1 at the tight junctions in the brains of animals at 24 h after ICH induction. ICH down-regulated the ZO-1 protein, and DPM reversed this to a significantly greater extent than PM or free DRC (Fig. 10a). Similar results were observed in western blots (Fig. 10b, c).

### Ability of DPM to inhibit ICH-induced apoptosis and ferroptosis

**Fig. 11:**
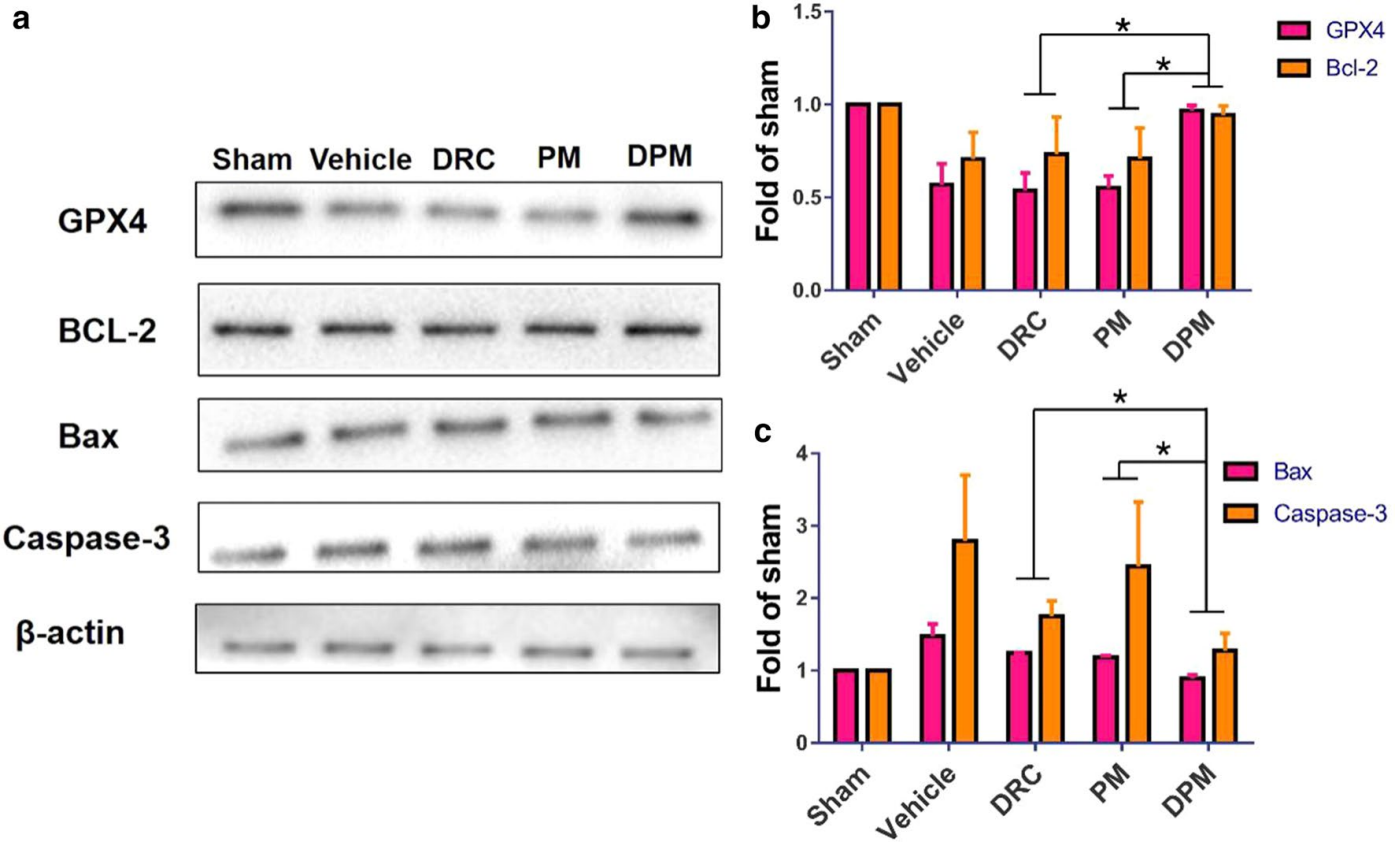
Effects of DPM on ICH-induced apoptosis and ferroptosis. **a** Representative Western blots of GPX-4, Bcl-2, Bax, and caspase-3 in the left hemisphere of at 24 h after ICH and treatment with nothing (sham), 0.9% saline (vehicle), free dauricine (DRC), empty micelles (PM) or dauricine-loaded micelles (DPM). Results are representative of three experiments. Levels of **b** GPX4 and Bcl-2 as well as **c** Bax and caspase-3 were quantitated relative to β-actin, and levels are expressed relative to that in the sham group. Values are mean ± SD, n = 3. *P < 0.05

The balance between Bcl-2 and Bax levels determines the balance between cell survival and death. While higher expression of Bcl-2 inhibits apoptosis, higher expression of Bax induces mitochondrial membrane permeabilization, release of cytochrome c, and activation of caspases-9 and -3, initiating apoptosis. Compared to sham surgery, ICH significantly decreased Bcl-2 expression and increased Bax and caspase-3 expression in the left hemisphere (Fig. 11a). DPM (100 mg/kg) reversed these effects, increasing the Bcl-2/Bax ratio (Fig. 11b, c). At the same time, DPM up-regulated glutathione peroxidase 4 (GPX4), and this enzyme down-regulates ferroptotic cell death by converting toxic lipid hydroperoxides into non-toxic lipid alcohols (Fig. 11b). PM and free DRC also up-regulated GPX4, although to a smaller extent.

### Ability of DPM to attenuate microglial activation, astrocyte activation, and neutrophil infiltration after ICH

**Fig. 12:**
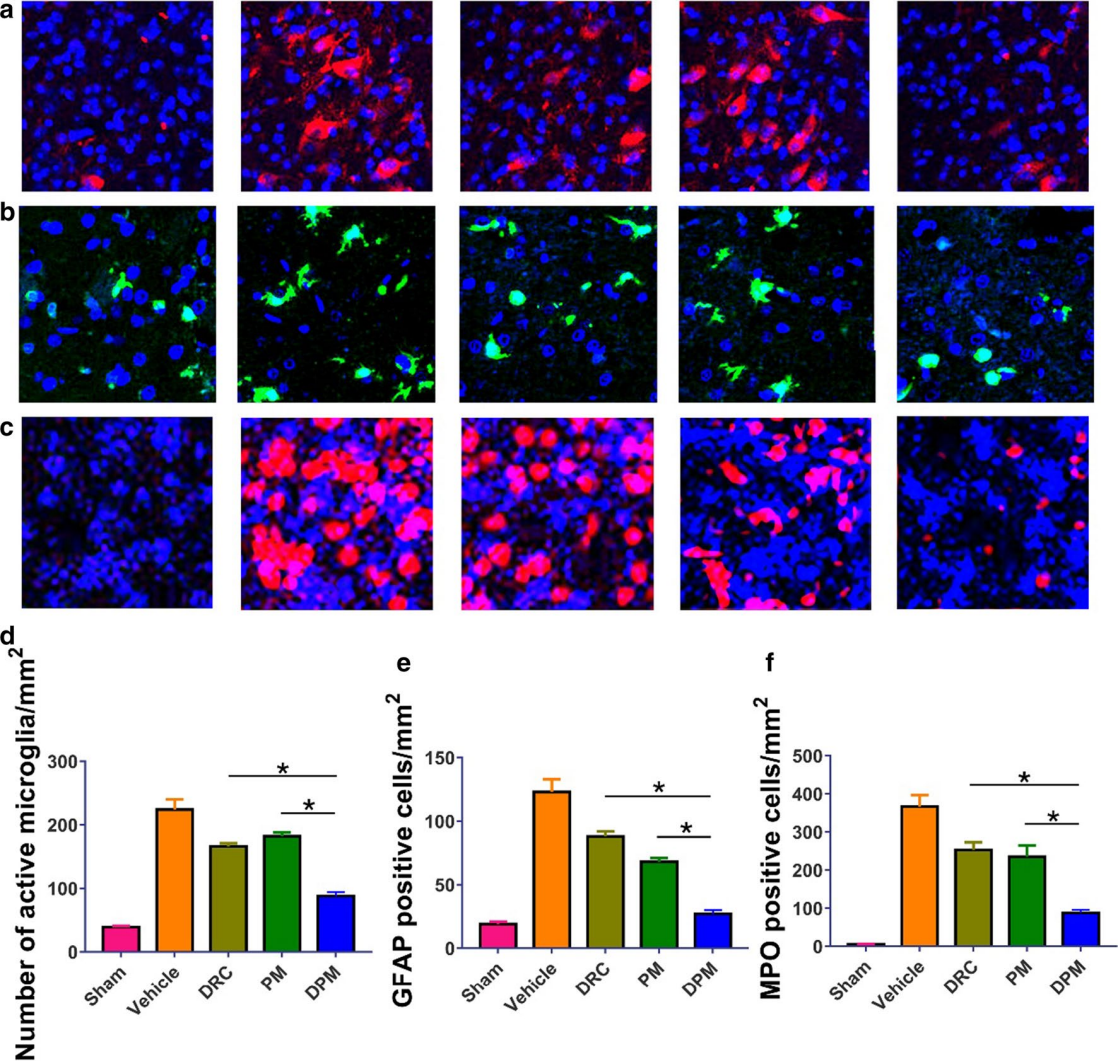
Ability of DPM to partially reverse ICH-induced activation of microglia and astrocyte as well as infiltration by neutrophils. Brain sections were prepared at 24 h after ICH and immunostained to detect the three cell populations. **a** Representative photomicrographs of brain tissue immunostained against Iba-1 to reveal microglia. Magnification, ×100. **b** Representative photomicrographs of brain tissue immunostained against GFAP to reveal astrocytes. Magnification, ×200. **c** Representative photomicrographs immunostained against MPO to reveal neutrophils. Magnification, ×100. **d–f** Quantitation of the experiments shown in panels (**a**–**c**), respectively. Values are mean ± SD, n = 3. *P < 0.05

To further examine additional mechanisms by which DPM might attenuate ICH injury, we assessed microglial activation, astrocytic activation, and neutrophil infiltration in perihematomal brain tissue at 24 h after ICH. Microglia were stained with Iba-1 immunofluorescence and their morphology examined: resting or quiescent microglia appear long, thin, and rod-like, while activated microglia have enlarged cell bodies with short processes. DPM led to significantly fewer activated microglia in perihematomal tissue (Fig. 12a). Astrocytic activation was assessed by observing GFAP-positive cells. DPM led to significantly less astrocytic activation in perihematomal tissue than free DRC or PM (Fig. 12b). Based on MPO staining, we found significantly fewer infiltrating neutrophils in the perihematoma of the DPM group than in the other groups (Fig. 12c).

### Ability of DPM to reduce ICH-induced production of IL-1β, IL-6, TNF-α and MMP-9

**Fig. 13:**
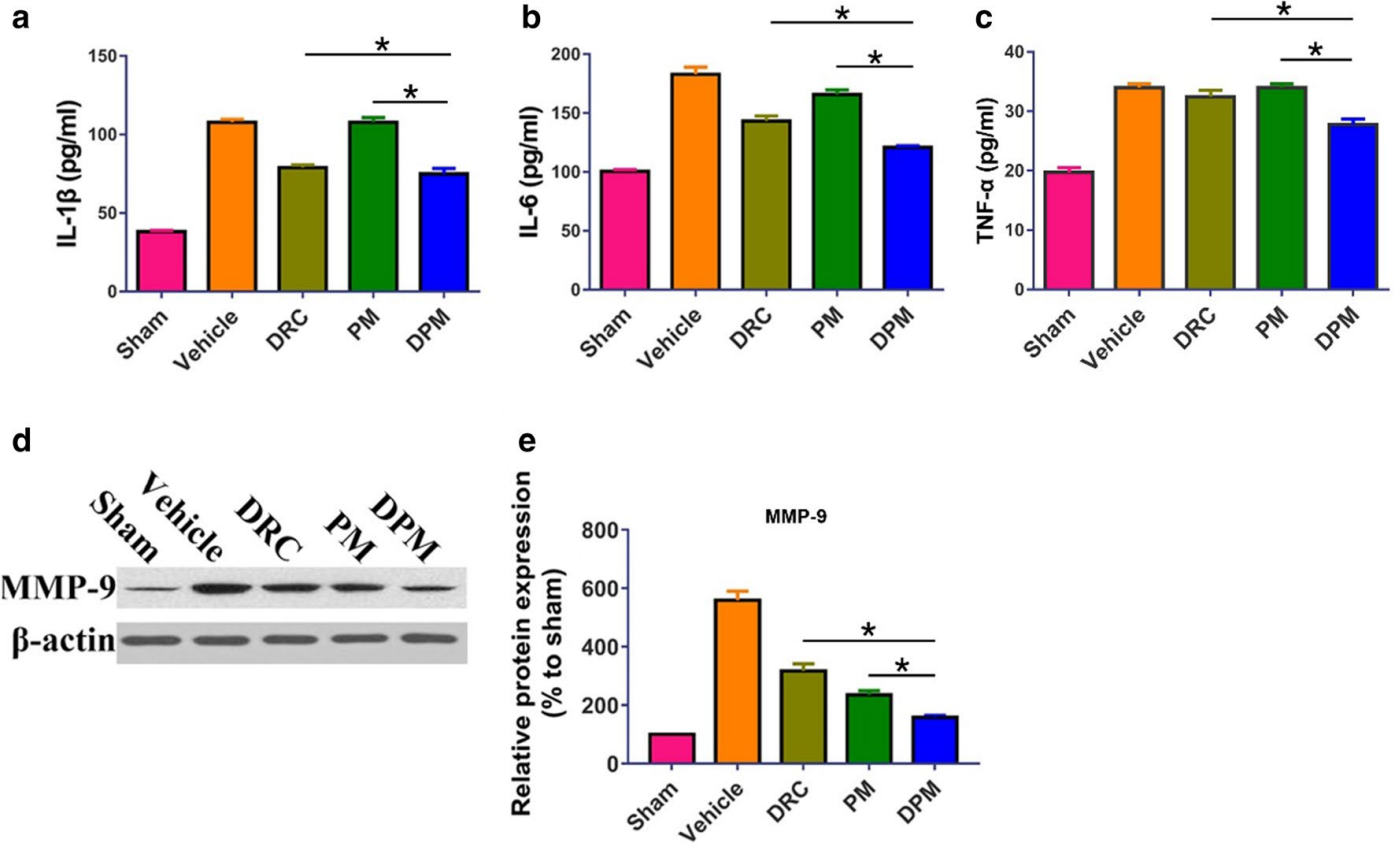
Effect of DPM on the ICH-induced pro-inflammatory state in the perihematomal area. Animals were subjected to ICH and treatment with 0.9% saline (vehicle), free dauricine (DRC), empty micelles (PM) or dauricine-loaded micelles (DPM). The sham group was set up by performing craniotomy without blood infusion. At 24 h later, brain tissue was isolated and assayed by ELISA for **a** IL-6, **b** IL-1β and **c** TNF-α, or by Western blot for **d**, **e** MMP-9. Values are mean ± SD (n = 3). *P < 0.05

To investigate the anti-inflammatory effects of DPM in ICH, the levels of pro-inflammatory factors IL-1β, IL-6, and TNF-α as well as MMP-9 in the perihematomal brain tissue were measured. As expected, ICH up-regulated the levels of all four factors, and DPM significantly reversed these effects (Fig. 13).

## Discussion

Intracerebral hemorrhage (ICH) occurs when a weakened vessel ruptures and bleeds into the surrounding brain [29, 30]. Various forms of cell death have been identified after ICH, including apoptosis, necrosis, and autophagy in humans and experimental animals, and autophagic cell death in animal models [31–33]. Although inhibiting apoptosis, necrosis, and autophagy can improve outcomes in animals subjected to experimental ICH, no successful clinical trials using cell-death inhibitor monotherapy have been reported [34–36]. These facts suggest that multiple forms of cell death other than those mentioned above may occur after ICH and contribute collectively to neuronal death.

Toxins released from an intracerebral hematoma may contribute to brain damage after ICH [37]. Two putative neurotoxins are hemoglobin, the most abundant protein in blood, and its heme group, which are released from lysed erythrocytes after ICH [38–40] Hemoglobin and its heme group play an essential role in ROS production after ICH [41–43]. In addition, hemoglobin can be metabolized into ferrous/ferric iron and form hydroxyl radicals via the Fenton reaction [44]. These highly toxic radicals attack DNA, proteins, and lipid membranes, leading to ferroptosis and production of pro-inflammatory factors [45, 46]. This recently described form of cell death plays a major role in tumor development and embryonic development and also contributes to neuronal death in a mouse model of ICH [47, 48].

These considerations suggest that effective ICH treatments should target the multiple injury pathways involved, including apoptosis, iron deposition and ferroptosis, ROS as well as inflammation [49, 50]. The present study shows that micelles assembled from a calix[4]arene scaffold with hydrophobic *n*-dodecyl chains can act as an Fe^2+^-responsive delivery vehicle for DRC to inhibit all of these ICH-induced damage pathways selectively at sites of brain injury. In our experiments, DPM decreased ROS levels *in vitro,* increased the Bcl-2/Bax ratio in brain tissues, and inhibited ferroptosis. These effects were associated with improvement in ICH-induced neurological defects. Our results suggest that DPM can alleviate primary and secondary brain injury driven by apoptosis and ferroptosis.

Among the macromolecules, calixarene is considered to represent the third-generation of host-guest supramolecular chemistry [51]. Calixarene and its derivatives have been reported to have antiviral, antibacterial, antifungal, antitubercular, and anticancer activity [52]. In the present work, from TEM images, we found that the size of PM and ad DPM are same as DLS. As we know Dynamic light scattering (DLS) was used to determine the hydrodynamic diameters of nanoparticles. Therefore, for inorganic nanoparticles, their DLS sizes are much higher than their TEM sizes, as the hydration shell around inorganic nanoparticles would evaporate after samples on TEM copper grid were dried under room temperature. However, cases would be different with organic nanoparticles, whose shape could change from 3D sphere to 2D thin film upon losing water outside and inside nanoparticles after drying under room temperature. In our previous research, we confirmed by atomic force microscopy (AFM) that nanoparticles would partially collapse, leading to about 20% larger size than that of DLS size [18]. Herein, for TEM images of PM and DPM, we care more about their morphology and sizes distribution. Similar discrepancy was observed by Yao Wang et al. who found that the size of Mac-1 with DOX from TEM images was around 30 nm in average, while most of the nanogel particles remained at smaller radius with the peak around 10 nm from DLS analysis [53].

Moreover, Calix[4]arene, bearing methylenebisphosphonic groups, can host cationic small drugs such as carboplatin or metal ions such as Cu^2+^ or Fe^2+^ in its cavity [10, 16, 17]. In previous work, we showed that micelles similar to PM in the present study delivered anti-cancer drugs in response to pH [54, 55]. In that study, the micelles had shorter *n*-hexyl chains, which we lengthened to *n*-dodecyl chains for this study to create larger micelles for higher drug loading. The present work further demonstrates the flexibility of the calix[4]arene scaffold for delivering drugs in response to specific stimuli. The micelles in the present study respond to high concentrations of metal cations. Since the pKa of the phosphonic acid head groups is 7.21, the head groups should be deprotonated at physiological pH of 7.4, and this charge may be neutralized by metal ions such as Fe^2+^ and Cu^2+^. Such neutralization may destabilize the micelles, accelerating release of the drug cargo. This may explain how the micelles can release drug preferentially in perihematomal tissue, where metal ion concentrations are elevated [56, 57].

And in our experiments, we chose 5 μM PM for further experiment.As shown in Fig. 3, optimized dose of DRC, PM and DPM was investigated by incubation of a serial of concentrations of DRC, PM and DPM with SH-SY5Y cells, with all treatment groups also receiving Fe^2+^ to a final concentration of 5 mM. 1 μM of DRC and DPM were chosen for further experiments since the discrepancy of cell viabilities is the hugest on this concentration. As calculated above, 1 μmol of DPM contains 5562.2 μg of *p*-PCa4C12, which equal to 3.8 μmol *p*-PCa4C12. To increase the redundancy, higher concentration (5 μM) of *p*-PCa4C12 micelles (PM) was used to compare with DPM for their treatment efficacies in ICH. Thus, 5 μM PM was correspondingly chosen for further experiment.

The reason why the concentrationof Fe^2+^ was determined in vitro is that in our preliminary experiment, toxicities of different concentrations of Fe^2+^ to SH-SY5Y cells after co-incubation for 24 h was evaluated, we found 5 mM Fe^2+^ could inflict 40% of cells death (Additional file 1: Figure S6). Higher concentration of Fe^2+^ as 10 mM would cause 55% cell deceased, which is too severe to be recovered by DPM. And lower concentration of Fe^2+^ as 1 mM would lead to 24% cell died, which is too mild to distinguish efficacies between DRC and DPM. Therefore, the concentration of 5 mM Fe^2+^ was deemed as appropriate concentration for further experiments. Cytotoxicity of Fe^2+^ varied significantly in different research groups. Some researcher group got similar result as what we got in terms of IC_50_ of Fe^2+^. Takahiko Imai et al. revealed 300 μM Fe^2+^ caused about 4% endothelial cells death after a 24 h incubation period, which translated to IC_50_ of 3.8 mM for Fe^2+^ [58]. Considering different lab’s conditions and cell lines, 5 mM Fe^2+^ inflicting 40% of cells death for in vitro experiments is plausible.

The FACS results in Fig. 5 show that there is only 15–17% apoptotic cells in the vehicle treated group, whereas the cell viability studies using CCk-8 shows much higher apoptosis, assuming at the same dose of 5 mM Fe^2+^ concentration. The main reason for the discrepancy between the results of cell viability and apoptosis is the incubation time is different. The incubation time for cell viability is 24 h, while the incubation time is only 12 h for apoptosis experiment. More importantly, in apoptotic test, after cultured for 12 h in the presence of 5 mM Fe^2+^ (to induce apoptosis) as well as 1 µM DRC, 5 µM PM or DPM (final DRC concentration, 1 µM), SH-SY5Y cells were incubated another 24 h to recover with fresh DMEM without any treatment or Fe^2+^.

DiR is a small hydrophobic molecule, which have shorter Tmax (time for peak of blood concentration) and then rather quickly excrete from kidney. In our research, DiR solution mainly accumulated in the liver, spleen and lungs after tail vein injection, which is consistent with literature [59]. Forming DiR loaded micelles usually would dramatically change its pharmacokinetic profiles, as shown in our in vivo results. After 24 h of tail vain injection, DiPM preferably accumulated in brain and lung, while its distribution reduced in other organs. The possible reasons for this phenomenon are as following: Firstly, DiPM would preferentially release DiR at sites of hematoma injury because the negative charges on the micelle surface would be neutralized by abundant metal ions in the surroundings. Furthermore, as shown in vitro releasing experiment (Fig. 2a), percentage of cumulative release will reach plateau after 24 h, which may explain higher accumulation of DiPM in 24 h than that in 12 h. Secondly, the accumulation of these DiPM in lung likely reflects their large size and persistence in systemic circulation. Consistent with other researcher has reported that particles < 20 nm are susceptible to clearance through kidneys while nanoparticles will continuous accumulated in the lung as their size increased [60]. Therefore, the intensity of fluorescence was higher in the lung in 24 h than that of 12 h. Finally, the fluorescent intensities of DiPM are negligible in either liver or spleen in both 12 h and 24 h, which may suggest DiPM could effectively escape the capture of RES (reticulo-endothelial system) in the liver and spleen. Thus, the concentrations of DiPM in both brain and lung were much higher than those in treatment group of DiR, considering much less accumulation of DiPM in other organs.

Blood–Brain Barrier (BBB) disruption is a hallmark of ICH-induced brain injury [61]. Therefore, after systemically administration of DPM, DPM will reach the hemorrhagic site after they pass through the ruptures in the BBB. Furthermore, after DPM chelating with metal ions including Fe(III), DPM may be further transferred across BBB by transferrin receptor 1, which is highly expressed by brain capillary endothelial cells (BCECs) [62, 63]. In the current research, DPM significantly reduced brain water content of both hemispheres at 24 h after ICH in our mouse model. Perihematomal edema includes cytotoxic edema due largely to apoptotic and ferroptotic cell death, and vasogenic edema resulting mainly from disruption of the blood–brain barrier [64]. ICH stimulates microglia and astrocytes, leading to production of inflammatory IL-1β and IL-6, which injure endothelial cells. MMP-9 is up-regulated, and it degrades the vascular matrix; while ZO-1 is down-regulated, weakening tight junctions [65]. All these changes compromise the integrity of the blood-brain barrier. At the same time, IL-1β causes endothelial cells to up-regulate adhesion molecules such as ICAM-1, VCAM-1, and E-selectin, which promote leukocyte margination and adhesion to the endothelial luminal surface, releasing proteases and cytokines and breaking the blood-brain barrier “from the outside” [66]. In our study, DPM significantly reduced the activation of microglia and astrocytes; inhibited neutrophil infiltration; down-regulated IL-1β, IL-6, TNF-α and MMP-9; and up-regulated ZO-1.

Following ICH, cytotoxic edema is accompanied by different forms of death of glial cells and neurons such as apoptosis and ferroptosis [67]. Previous studies have suggested that apoptosis is the main mechanism of early tissue injury in the region surrounding the hematoma after ICH [68]. Many factors induce cell apoptosis after ICH, such as free radical cascade reactions, inflammation, cytokine stimulation, and induction of thrombin and blood components. In the present study, DPM decreased ROS levels in vitro, thereby alleviating oxidative stress and stopping cascade reactions. Consistent with previous findings, we found that ICH decreased the ratio of Bcl-2/Bax in brain tissues, which was reversed by DRC and DPM, corresponding to less apoptosis of neurons. Since ferroptosis is regarded as one of the main causes of secondary brain injury, reducing it further improves neurological functional outcomes following ICH. We observed that PM and DPM also significantly attenuated ferroptosis of neurons after ICH.

Ferroptosis is a form of regulated cell death characterized by the iron-dependent accumulation of lipid hydroperoxides to lethal cellular levels [69]. GPX4 converts potentially toxic lipid hydroperoxides (L-OOH) to non-toxic lipid alcohols (L-OH). Free iron inhibits the regeneration of glutathione via the Fenton reaction, which inactivates GPX4 and ferroptosis [70]. In the present study, we found that both PM and DPM significantly preserved GPX4, probably by chelating metal ions such as Cu^2+^ and Fe^2+^, thereby alleviating ferroptosis. Therefore, by simultaneously preventing apoptosis and ferroptosis, DPM may alleviate both early and secondary brain injury for ICH.

## Conclusion

Our studies in a mouse model suggest that DPM can protect against ICH-induced disruption of the BBB, brain edema and neurological deficits. The formulation appears to work by inhibiting ICH-induced activation of neuroglia, infiltration by neutrophils, production of pro-inflammatory factors (IL-1β, IL-6, TNF-α) and MMP-9, and down-regulation of the tight junction protein ZO-1. The ability of DPM to target both apoptosis and ferroptosis as well as weaken the pro-inflammatory state after ICH may make it an effective therapy.

## Supplementary information

**Additional file 1.** Additional figures.

## Data Availability

Data and materials are available upon request. The raw/processed data required to reproduce these findings cannot be shared at this time as the data also forms part of an ongoing study.
