# Supplementary material for: Metal ion-responsive nanocarrier derived from phosphonated calix[4]arenes for delivering dauricine specifically to sites of brain injury in a mouse model of intracerebral hemorrhage
Source: J Nanobiotechnology. 2020 Apr 19;18:61. doi: 10.1186/s12951-020-00616-3 (PMC7168846; doi:10.1186/s12951-020-00616-3)
Supplement: Supplementary file 1 — Additional file 1. Additional figures. [file 12951_2020_616_MOESM1_ESM.docx]

**Metal ion-responsive nanocarrier derived from phosphonated calix[4]arenes for delivering dauricine specifically to sites of brain injury in a mouse model of intracerebral hemorrhage**

Mingxin Li, Guohao Liu, Kaixuan Wang, Lingfeng Wang, Xiang Fu, Lee Yong Lim, Wei Chen *, Jingxin Mo *

**Synthesis of compound *p*-PCa4C12**

*p*-PCa4C12 was synthesized as described previously.[[1-3](#_ENREF_1)] Briefly, *n*-hexyl groups were attached to the lower rim of calix[4]arene via Duff reaction-enabled formylation with bromohexane and sodium hydride in DMF, and the formylated compound was reduced to alcohol on the upper rim of calix[4]arene using sodium borohydride. The alcoholic group was chlorinated by thionyl chloride, phosphorylated by triethylphosphite and finally deprotected by bromotrimethylsilane. The chemical structure of the resulting *p*-PCa4C12 after purification by silica gel column was confirmed by ^1^H-NMR (Mercury 400, Varian, Palo Alto, CA; **Figure** S1).
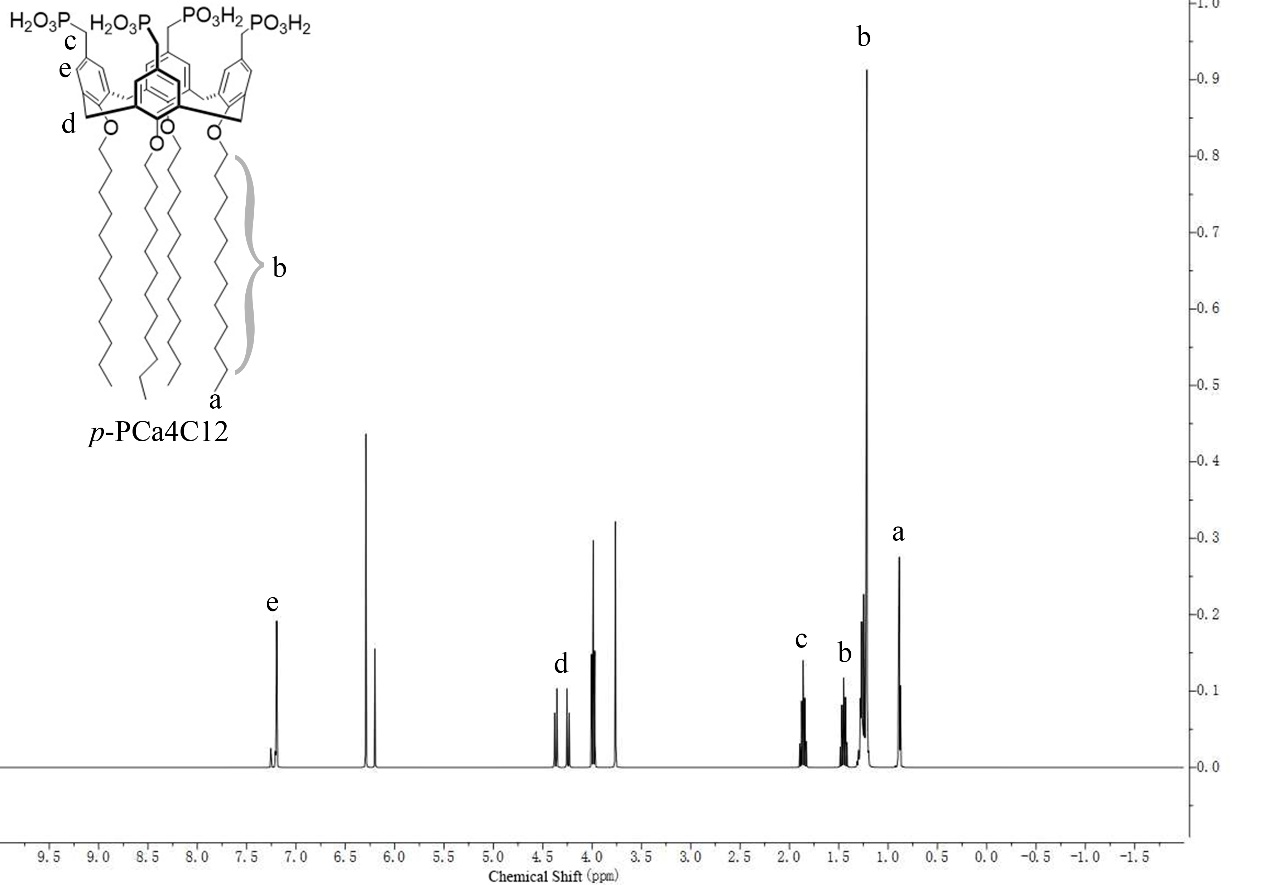


**Figure** S1. ^1^H-NMR spectrum of *p*-PCa4C12.

**Mouse model of ICH**


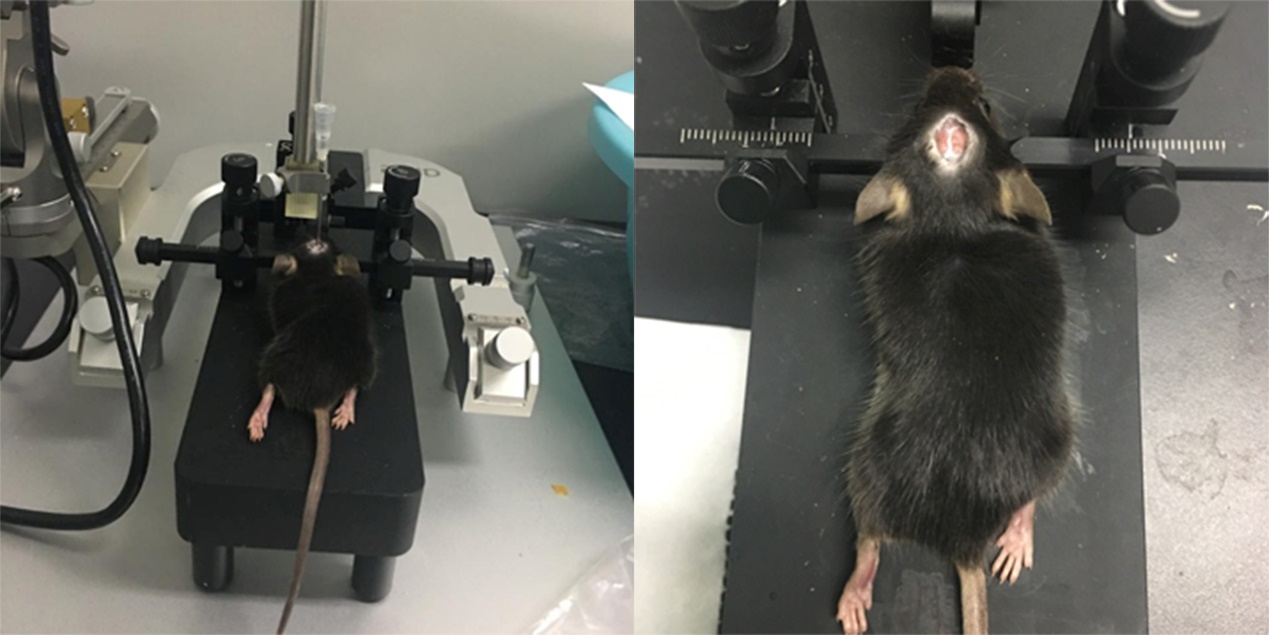


**Figure** S2. Images showing a C57BL/6 mouse being prepared for ICH induction.

**Determination of DPM encapsulation efficiency and drug loading**

Liquid chromatography was performed on a Shimadzu LC-20A HPLC system (Shimadzu, Shanghai, China). Dauricine (DRC) was separated on an ODS C_18_ column (150 × 4.6 mm, 5 μm; Inertsil, Shimadzu) under isocratic elution at a flow rate of 1 mL/min. The mobile phase consisted of acetonitrile : 0.1% phosphoric acid triethylamine solution (20:80, v/v). Dauricine was monitored using an SPD-20AV ultraviolet spectrophotometer. The UV spectrometer was operated between 200-400 nm with 280 nm as maximum absorption wavelength for DRC. Injection volume was 10 µL.[[4](#_ENREF_4)]

Freeze-dried DPM powder (0.5 mg) was precisely weighed, dissolved in 2 mL of mobile phase, and vibrated vigorously for 2 mins. The mixture was centrifuged for 10 min at 6037 *g,* and the supernatant was filtered (0.22 μm, Millipore) and injected into the HPLC system.

Encapsulation efficiency (EE) and drug loading (DL) were calculated using the following formulas:

EE (%) = (amount of encapsulated drug / initial amount of drug) × 100 %.

DL (%) = (amount of encapsulated drug) / (amount of DPM powder) × 100 %


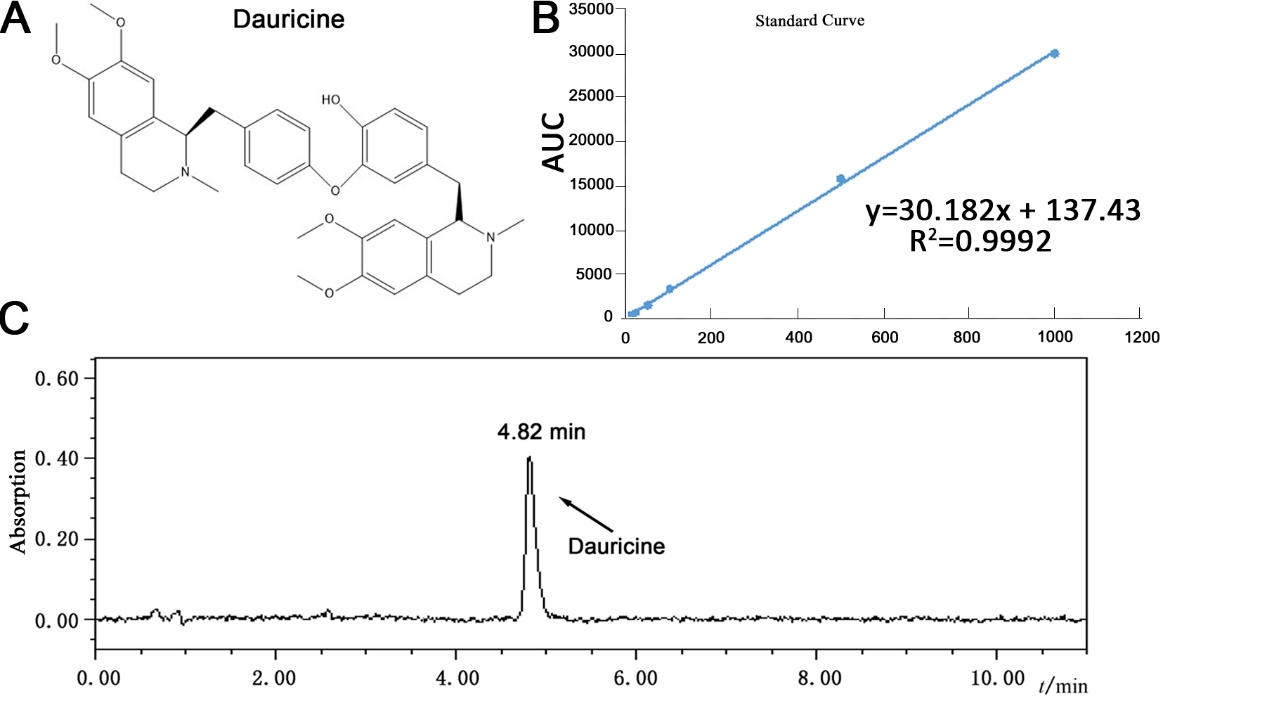


**Figure** S3. **(A)** Molecular formula of dauricine (DRC), **(B)** standard DRC curve, and **(C)** typical chromatogram by ultraviolet spectrophotometry on HPLC.

**Quantification of DPM by HPLC assay**

As shown in **Figure** S3C, the retention time for DRC was 4.82 min. Calibration curves of DRC (y = 30.182x + 137.43, R² = 0.9992, **Figure** S3B) exhibited excellent linearity over the concentration range from 10 to 2,000 ng/mL. High, medium and low concentrations of intra- and inter-day precision RSD were less than 2.0%, indicating compliance with the established LC-UV assay requirements.

***In vitro* stability tests**

Freshly prepared DPM (1 mg) was incubated in 10 mL of 0.9% saline at 37 °C. At different time points, the particle size distribution profile, polydispersity index (PDI) and zeta potential were characterized by dynamic light scattering.


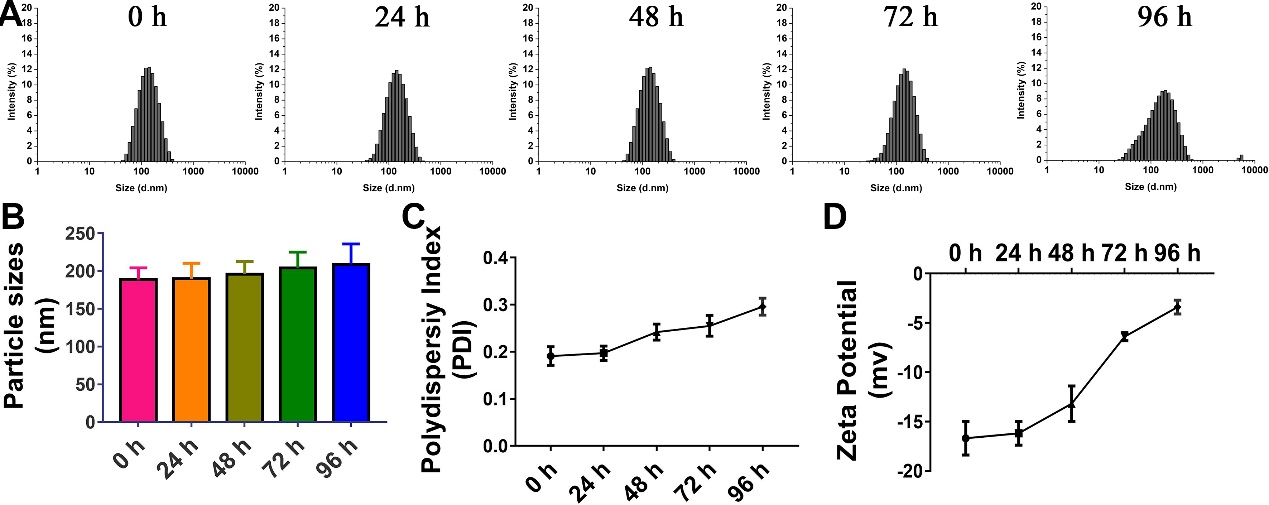


**Figure** S4. Measurements of DPM dispersions in 0.9% saline at 0, 24, 48, 72 and 96 h. **(A)** Particle size distribution, **(B)** Mean particle size, **(C)** Polydispersity index and **(D)** zeta potential. Error bars indicate SD (n = 3).

**Figure** S4 shows no significant changes in mean particle size upon incubation for 96 h. However, the polydispersity index and zeta potential increased with longer incubation, indicating that the DPM after reconstitution in 0.9% saline is unstable and should be prepared immediately before use or lyophilized for storage.

**Neuroprotective Effects of Nanoparticle Formulations**


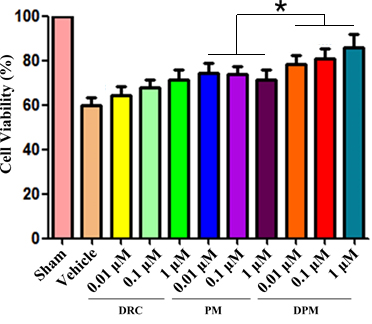


**Figure** S5 Neuroprotective effects of various formulations on SH-SY5Y cells incubated with 600 µM Cu^2+^. The cells were treated with a series of concentrations of free DRC, PM or DPM.

Figure S5 shows that even low concentrations of DPM substantially reduced the toxicity of Cu^2+^ on SH-SY5Y cells.

**Toxicity of Ferrous ion to SH-SY5Y cells**


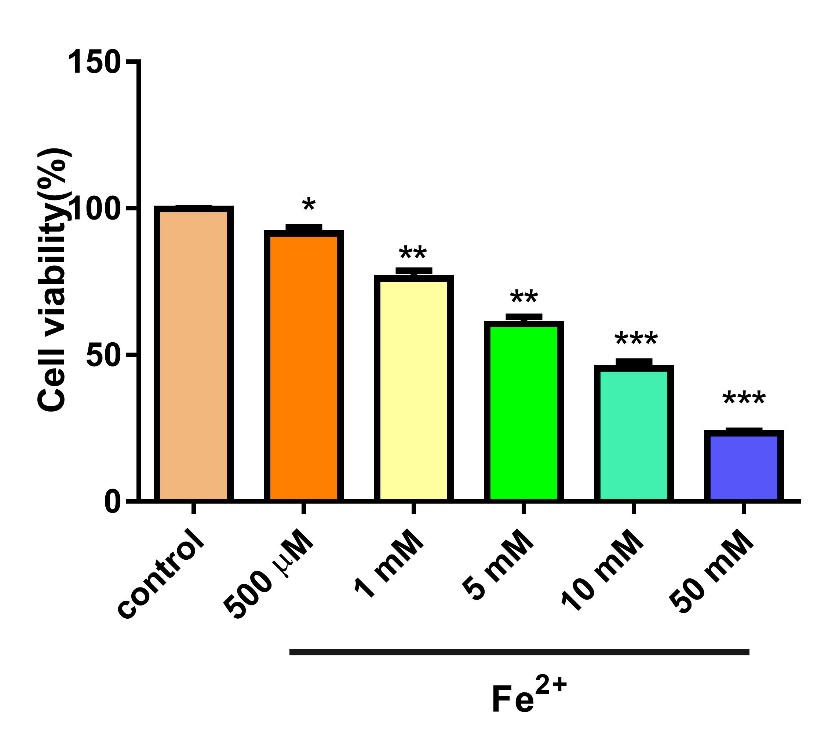


**Figure** S6. Toxicity of a series concentration of ferrous ion on SH-SY5Y cells after co-incubation for 24 h. *P<0.05, **P<0.01, ***P<0.001. (n=5)

As shown in figure S6, the toxicity of ferrous ion to SH-SY5Y cells is dose-dependent with IC_50_ is about 6 mM after 24 h incubation.

**Toxicity of empty *p*-phosphonated calix[4]arene micelles (PM) to SH-SY5Y cells**


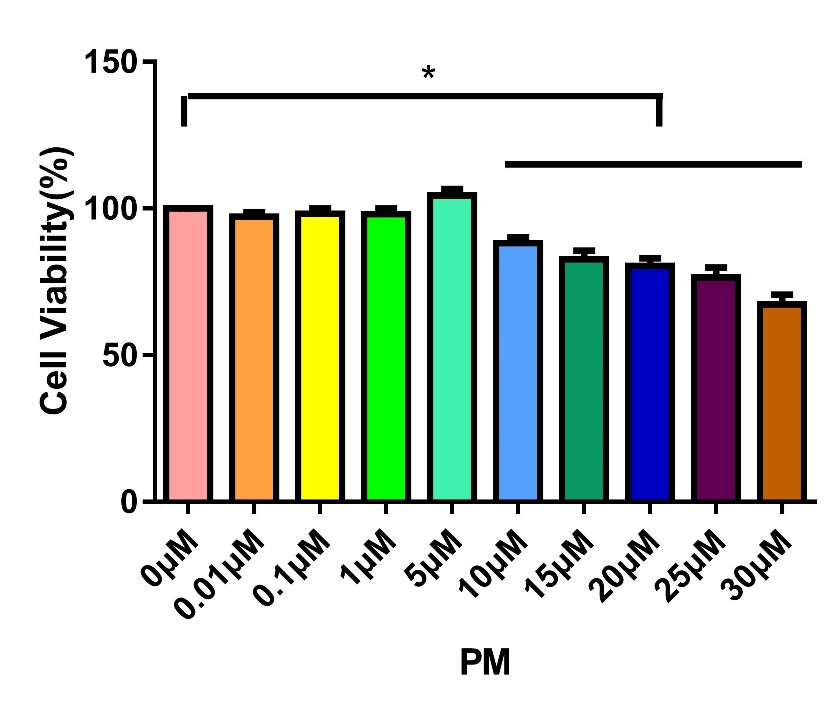


**Figure** S7. Toxicity of a series concentration of PM on SH-SY5Y cells after co-incubation for 24 h. *P<0.05. (n=5)

As shown in figure S7, PM showed good tolerance with SH-SY5Y. It would cause damage to the cells only the concentration exceed 10 μM after 24 incubation.

**Neurofunctional assessments**

**Paw placement test**

Mice were evaluated for their ability to respond to a whisker-elicited excitation, by forward movement of their paw. To execute this test, animals were held by the trunk, ensuring that the forelimbs were not restrained. The rodent was then oriented in parallel to a table top and slowly moved vertically until the whiskers on one side touched the surface.[[5](#_ENREF_5), [6](#_ENREF_6)] Adequate forward movements of the ipsilateral paw in response to whisker stimulation were assessed in 10 trials.

**Corner turn test (CTT)**

The CTT was performed as previously described. Mice were allowed to proceed into a 30° corner. To exit the corner, animals had to turn right or left, typically by rearing along the corner wall. [[7](#_ENREF_7)]This was repeated 10 times, with at least 30 s between trials. The score was then calculated using the following formula: (Number of right turns / all turns) ×100%. Only turns involving a full rearing along either wall were recorded.


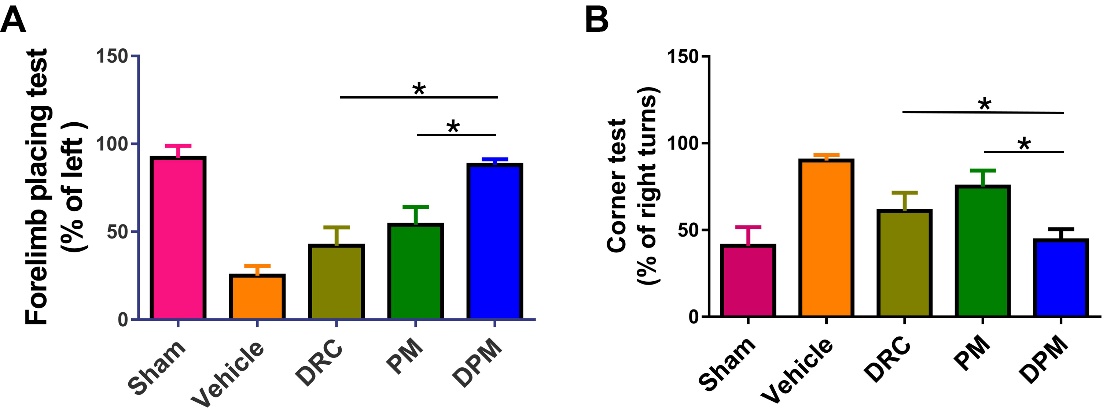


**Figure** S8. Statistical analysis of behavioral outcomes. Mice were injected in the tail vein with 0.9% saline, DRC, PM or DPM. Effects of DPM on **(A)** forelimb placing test and the **(B)** corner turn test at 24 h after intracerebral hemorrhage induction or sham surgery. Results are expressed as mean ± SD of sham performance (n = 10 in each group). *p<0.05.

The paw placement and corner tests were performed at 24 h after induction of ICH and DPM treatment to examine the behavioral changes. The animals with ICH had significantly lower paw placement scores than sham-operated animals (**Figure** S8A). After treatment with DPM, ICH-induced paw placement defects were significantly less severe than in the other treatment groups. ICH markedly increased the frequency of right turns, which DPM reversed (**Figure** S8B).

References:

[1] Mo J, Eggers PK, Raston CL, Lim LY. Development and validation of a LC/TOF MS method for the determination of carboplatin and paclitaxel in nanovesicles. Analytical and bioanalytical chemistry,2014,406,2659-2667.

[2] Mo J, Eggers PK, Yuan ZX *et al.* Paclitaxel-loaded phosphonated calixarene nanovesicles as a modular drug delivery platform. Scientific reports,2016,6,23489.

[3] Chen W, Li L, Zhang X *et al.* Curcumin: a calixarene derivative micelle potentiates anti-breast cancer stem cells effects in xenografted, triple-negative breast cancer mouse models. Drug delivery,2017,24,1470-1481.

[4] Wei J, Fang L, Liang X *et al.* A sensitive and selective UPLC-MS/MS method for simultaneous determination of 10 alkaloids from Rhizoma Menispermi in rat plasma and its application to a pharmacokinetic study. Talanta,2015,144,662-670.

[5] Quattromani MJ, Hakon J, Rauch U *et al.* Changes in resting-state functional connectivity after stroke in a mouse brain lacking extracellular matrix components. Neurobiology of disease,2018,112,91-105.

[6] Zhou F, Jiang Z, Yang B, Hu Z. Magnolol exhibits anti-inflammatory and neuroprotective effects in a rat model of intracerebral haemorrhage. Brain, behavior, and immunity,2019,77,161-167.

[7] Krafft PR, McBride DW, Lekic T *et al.* Correlation between subacute sensorimotor deficits and brain edema in two mouse models of intracerebral hemorrhage. Behavioural brain research,2014,264,151-160.
